# Differential effects of FcRn antagonists on the subcellular trafficking of FcRn and albumin

**DOI:** 10.1172/jci.insight.176166

**Published:** 2024-05-07

**Authors:** Guanglong Ma, Andrew R. Crowley, Liesbeth Heyndrickx, Ilse Rogiers, Eef Parthoens, Jolien Van Santbergen, Raimund J. Ober, Vladimir Bobkov, Hans de Haard, Peter Ulrichts, Erik Hofman, Els Louagie, Bianca Balbino, E. Sally Ward

**Affiliations:** 1 Centre for Cancer Immunology, Faculty of Medicine, University of Southampton, Southampton, United Kingdom.; 2 argenx, Zwijnaarde, Ghent, Belgium.; 3 VIB BioImaging Core, Center for Inflammation Research, Ghent, Belgium.

**Keywords:** Autoimmunity, Immunology, Autoimmune diseases, Immunoglobulins

## Abstract

The homeostasis of IgG is maintained by the neonatal Fc receptor, FcRn. Consequently, antagonism of FcRn to reduce endogenous IgG levels is an emerging strategy for treating antibody-mediated autoimmune disorders using either FcRn-specific antibodies or an engineered Fc fragment. For certain FcRn-specific antibodies, this approach has resulted in reductions in the levels of serum albumin, the other major ligand transported by FcRn. Cellular and molecular analyses of a panel of FcRn antagonists have been carried out to elucidate the mechanisms leading to their differential effects on albumin homeostasis. These analyses have identified 2 processes underlying decreases in albumin levels during FcRn blockade: increased degradation of FcRn and competition between antagonist and albumin for FcRn binding. These findings have potential implications for the design of drugs to modulate FcRn function.

## Abstract 2

The use of FcRn antagonists to treat antibody-mediated disease is rapidly expanding, and antagonist design is relevant to differential effects on albumin recycling via effects on two processes.

## Introduction

The neonatal Fc receptor (FcRn) is an MHC class I–related heterodimer composed of a heavy chain (FcRn-α) in complex with β2 microglobulin (β2m) (1). This widely expressed receptor binds to both IgG and albumin in a pH-dependent manner (2–8). Sites for IgG and albumin on FcRn encompass distinct residues and do not overlap, allowing both ligands to interact with this receptor simultaneously (6, 8–10). The pH dependency enables FcRn to preferentially bind its ligands in the mildly acidic (pH 6.0–6.5) environment of early endosomes and selectively salvage its bound cargo from lysosomal degradation (11–14). Following return to the extracellular environment through transport by tubulovesicular carriers recycling to the cell surface, or transcytosing to the opposing face of a polarized cell, the FcRn-ligand affinity becomes negligible at the extracellular pH of 7.3–7.4 and cargo is released (15–19). In this manner, FcRn mediates several critical aspects of humoral immunity. For example, expression of FcRn in placental syncytiotrophoblasts facilitates transport of maternal IgG from mother to fetus, providing an important source of humoral protection to newborns during their first months (20–22). FcRn expression in hematopoietic, endothelial, and epithelial cells also regulates IgG and albumin levels and transport throughout life (23–28). The highly active internalization of IgG and albumin via fluid phase pinocytosis or macropinocytosis in hematopoietic cells such as macrophages results in FcRn-mediated salvage in these cells being particularly important for maintenance of levels of the 2 proteins (26–29).

The contribution of FcRn to regulating the half-life and transport of IgG has prompted antibody engineering efforts to modulate IgG persistence (30). For example, improving the affinity of IgG for FcRn under acidic conditions while retaining negligible affinity at physiological pH generates antibodies capable of persistence in the body for a significantly longer time than those with a WT Fc region (31–33). However, in autoimmune disorders mediated by pathogenic, autoreactive IgG antibodies, increased clearance is instead desirable (34–36). This has motivated the development of molecules capable of antagonizing FcRn with the goal of reducing total IgG levels, including pathogenic autoantibodies (37). These antagonists may be grouped into 2 categories. The more common approach is generating monoclonal antibodies (mAbs) using the conventional antigen recognition activity of their antigen-binding fragments (Fabs) to bind FcRn via variable domains with high, similar affinity across the physiological pH range 6.0–7.4 with the goal of inhibiting IgG-FcRn binding (38–40). These antagonists bind to residues on FcRn that do not directly overlap with the Fc-FcRn interaction site (41, 42). In contrast, the so-called Abdeg technology, for antibodies that enhance IgG degradation, represents an alternative strategy for FcRn antagonism involving the substitution of 5 IgG1 Fc residues (M252Y, S254T, T256E, H433K, N434F; “MST-HN’’) that are located at the site of FcRn binding on the Fc fragment of a human IgG1 molecule (43). These mutations increase the affinity of the Fc-FcRn interaction at both acidic and neutral pH while docking at the “natural” binding site and retaining the intrinsic pH dependency (43). In late 2021, efgartigimod, an engineered human Fc fragment based on Abdeg technology, received FDA approval in the United States for the treatment of generalized, acetylcholine receptor antibody–positive myasthenia gravis after demonstrating ability to ameliorate disease in parallel to reducing levels of acetylcholine receptor–specific IgGs, followed by approvals in Japan and the European Union (44, 45). The more recent approval of the mAb rozanolixizumab for generalized, acetylcholine receptor– and muscle-specific tyrosine kinase antibody–positive myasthenia gravis has expanded the range of drugs acting via FcRn antagonism (46, 47).

The diverse roles and multiple ligands of FcRn need consideration when evaluating therapeutic antagonists targeting this receptor, as alterations in the homeostasis of serum albumin in parallel with the desired reductions in IgG levels have been associated with their use. For example, during a phase I trial, the mAb nipocalimab demonstrated a transient decline in albumin (3.6 to 2.8 g/dL) in patients receiving 30 mg/kg weekly doses for 4 weeks (40), and this transient decline in albumin level was observed in another single-dose phase I study, ranging from 4.4% to 8.2% for 30 mg/kg infusion cohorts and 14.6% for the 60 mg/kg infusion cohort (48). In a phase II clinical trial using another mAb, batoclimab, for treatment of myasthenia gravis, serum albumin declined in a dose-dependent fashion before returning to normal levels 6 weeks after discontinuation of the study drug. The mean maximum decreases were 23.1% (6 weekly 340 mg doses cohort) and 32.9% (6 weekly 680 mg doses cohort) (49). By contrast, slight, transient increases in albumin levels, which remained within the normal range, were observed upon treatment of patients with pemphigus vulgaris by efgartigimod, an engineered Fc fragment (25 mg/kg weekly doses cohort), over 34 weeks during a phase II trial (50).

As the most abundant protein found in serum, albumin fulfills multiple roles that are at risk of perturbation if the concentration of this protein is not within the normal range. The functions of albumin include maintenance of oncotic pressure in blood vessels, transport of numerous endogenous molecules including fatty acids, and scavenging of free radicals (51–54). Exogenous compounds may also readily bind albumin, and in the case of certain drugs, particularly those with a narrow therapeutic index, their efficacy versus potential for toxicity may depend on albumin homeostasis (55–60). The linkage between serum albumin and lipid metabolism could explain why increases in LDL have been observed following FcRn antagonism (55, 61, 62). Indeed, declining albumin was the probable cause of an LDL rise that necessitated halting a phase II trial of batoclimab for treatment of thyroid eye disease (63–65). This situation may be considered analogous to hypercholesterolemia that frequently presents among patients with a rare congenital form of analbuminemia (66), an outcome also recapitulated in an albumin-deficient mouse model (67).

Each of the FcRn antagonists, regardless of whether the mode of action is Fc or Fab arm based, is directed toward the same functional outcome, to outcompete endogenous IgG for binding to the interaction site for this ligand on FcRn, increasing lysosomal delivery of IgG and degradation. Differential effects of FcRn antagonists on serum albumin levels have been reported in clinical trials. Although these observations are currently not well understood, it is likely that the specific characteristics of drug design play a role. In the current study, we present the results of an investigation into the possible mechanisms underpinning dysregulation of albumin homeostasis in the presence of different FcRn antagonists. A complementary approach comprising cellular, molecular, and in vivo analyses has been used to investigate how FcRn antagonists with differing epitopes, sizes, and modes of action influence albumin dynamics. Our results suggest that at least 2 distinct mechanisms can account for decreased serum albumin levels, namely accelerated FcRn degradation and, for one of the antagonists, steric hindrance of the albumin binding site on FcRn.

## Results

### Recombinant FcRn antagonists have expected effects on IgG recycling.

The panel of FcRn antagonists used in the current study consisted of 2 full-length human IgG1 antibodies specific for FcRn, HL161BK, and N027, i.e., analogs of batoclimab and nipocalimab (41, 42); ARGX-113, an efgartigimod analog (68, 69); and as controls, an IgG1-derived Fc fragment (Fc-IgG1-WT) and a full-length IgG1 (IgG1-WT) (70) (Supplemental Figure 1A; supplemental material available online with this article; https://doi.org/10.1172/jci.insight.176166DS1). Size-exclusion chromatography indicated that the protein preparations contained less than 1% aggregated material following purification (Supplemental Figure 1B).

Consistent with their mechanism of action, all FcRn antagonists reduced recycling of IgG in HEK293 cells stably transfected with human FcRn (hFcRn)-GFP and β2m (HEK293-hFcRn-GFP) during pulse-chase experiments followed by evaluation using flow cytometry. In the absence of FcRn antagonists, the percentage of maximum residual Alexa Fluor 647–labeled human IgG (hIgG-AF647) following the chase was approximately 20%, while in the presence of FcRn antagonists, the levels of residual hIgG-AF647 following the chase were approximately 80%–90%, indicating substantially reduced recycling of IgG (Supplemental Figure 1, C–E).

### Differential effects on FcRn levels in cells treated with FcRn antagonists.

**Figure 1:**
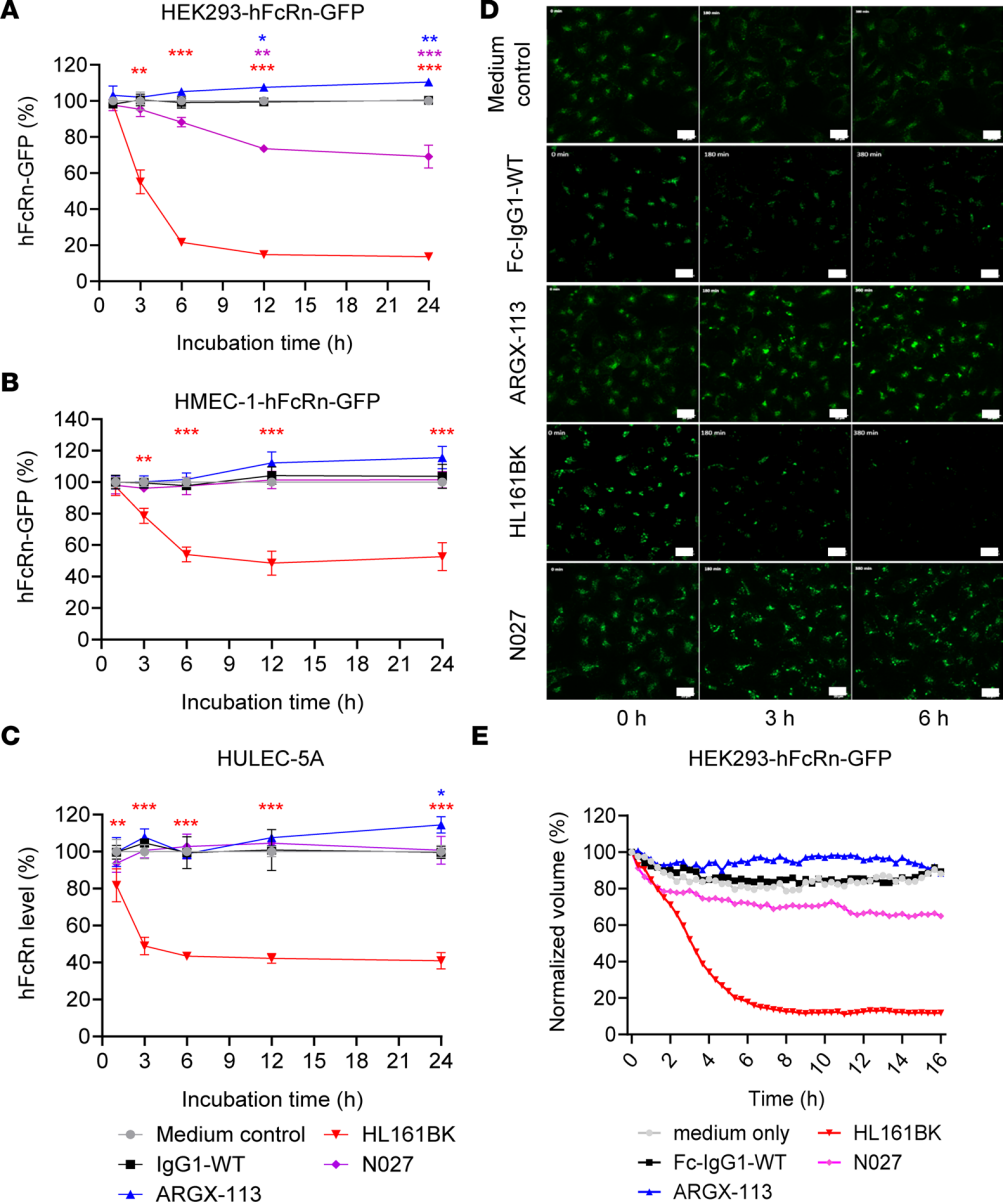
Analyses of hFcRn levels in FcRn antagonist–treated cell lines. hFcRn-expressing cell lines were incubated with 50 nM FcRn antagonist or medium alone (as a control) for the indicated times. The levels of GFP fluorescence were determined using flow cytometry in stably transfected HEK293-hFcRn-GFP (**A**) and transiently transfected HMEC-1-hFcRn-GFP (**B**) cells. Endogenous hFcRn levels in HULEC-5A cells (**C**) were assessed by fixing and permeabilizing the cells before staining with a fluorescently labeled Fab fragment specific for FcRn (Synt002-Fab-AF647) and determining AF647 levels using flow cytometry. At each time point, the MFIs are normalized to the corresponding medium control. These data are combined from 2 independent experiments, with triplicate samples in each experiment. Statistical analysis was performed with a linear mixed model, and significant differences compared with medium control are denoted: *P ≤ 0.05, **P ≤ 0.01, ***P ≤ 0.001. Error bars indicate the standard deviation of the mean. GFP levels over the 16-hour incubation with 500 nM of each FcRn antagonist were also monitored in HEK293-hFcRn-GFP cells using live-cell microscopy on a confocal spinning disk system (ZEISS). (**D** and **E**) GFP fluorescence is presented as maximum intensity projections at 0, 3, and 6 hours (**D**) and as normalized volume (sum of voxels) relative to *t_0_* (**E**). The data shown for the live-cell imaging are representative of 3 independent experiments. GFP is pseudocolored green. Scale bars = 20 μm.

HEK293-hFcRn-GFP cells were incubated with FcRn antagonists (i.e., HL161BK, N027, ARGX-113), IgG1-WT, or a medium-only control at time points up to 24 hours. Subsequently, the MFI of the GFP signal was used as an indicator of levels of GFP-tagged hFcRn. HL161BK-treated HEK293-hFcRn-GFP cells showed decreased GFP fluorescence over time, with greater than 80% of the GFP signal being lost following 24 hours of treatment. N027-treated cells also showed a decrease in the amount of GFP fluorescence, with an approximately 30% reduction in signal after 24 hours (Figure 1A and Supplemental Figure 2A). Similar effects on hFcRn-GFP downregulation were observed using a range of antagonist concentrations (50–500 nM), though a concentration of 5 nM did not induce maximal downregulation for HL161BK or N027 (Supplemental Figure 2B). In contrast with effects of HL161BK and N027, cells treated with ARGX-113 showed an increase (approximately 10%) in GFP signal following 24 hours of incubation (Figure 1A). To exclude the possibility that the size difference between the ARGX-113 Fc fragment and the full-length antibody antagonists was responsible for these observations, HEK293-hFcRn-GFP cells were also treated with a full-length IgG1 of irrelevant antigen specificity (i.e., hen egg lysozyme specific), which contains the same FcRn-enhancing mutations as ARGX-113, termed IgG1-MST-HN (43). No significant differences in GFP levels were observed between treatment with IgG1-MST-HN or ARGX-113 (Supplemental Figure 2A).

The analyses described above were carried out using medium supplemented with FBS depleted of IgG. To ensure that absence of IgG in the medium had not influenced the results, we carried out experiments using medium containing the ligands for FcRn, human serum albumin (HSA) and hIgG, in HEK293-hFcRn-GFP cells. Presence of HSA and hIgG in the medium did not influence FcRn downregulation (Supplemental Figure 2C).

To assess the generality of these observations, we investigated the effects of the FcRn antagonists on hFcRn-GFP levels in human dermally derived endothelial cells (HMEC-1) (12, 71). Transient transfection (hFcRn/β2m) of HMEC-1 cells (HMEC-1-hFcRn-GFP) resulted in a range of hFcRn-GFP expression levels that were lower than those present in stably transfected, clonal HEK293-hFcRn-GFP cells (compare the MFI levels for hFcRn-GFP in Supplemental Figure 2, A and D). Untreated cells were used to define the hFcRn^+^ HMEC-1 population. Consistent with the observations in HEK293-hFcRn-GFP cells, treatment with HL161BK led to the downregulation of hFcRn-GFP, with a 50% decrease in GFP fluorescence levels compared with medium-treated cells following a 24-hour incubation (Figure 1B and Supplemental Figure 2D). However, in contrast with results obtained using HEK293-hFcRn-GFP cells, N027-treated HMEC-1-hFcRn-GFP cells did not show a significant change in GFP fluorescence levels at any time point. An increase in GFP fluorescence was observed in HMEC-1-hFcRn-GFP cells following treatment with ARGX-113, analogous to that seen in HEK293-hFcRn-GFP cells, though the increase was not statistically significant (*P* > 0.05). Along with the level of hFcRn-GFP, the changes in the hFcRn^+^ population in HMEC-1-hFcRn-GFP cells were investigated. The FcRn^+^ population decreased with HL161BK treatment and to a greater extent than that for other antagonists. Following 24-hour incubation, less than 10% of HL161BK-treated cells were hFcRn-GFP^+^, whereas with other treatments over 30% of GFP^+^ cells were retained (Supplemental Figure 2E). As with HEK293-hFcRn-GFP cells, no significant difference in GFP fluorescence was observed in HMEC-1-hFcRn-GFP cells treated with IgG1-MST-HN compared with those treated with ARGX-113 (Supplemental Figure 2, D and E). In addition, immunoblotting analyses indicated that endogenous FcRn levels in HMEC-1-hFcRn-GFP cells were reduced by HL161BK treatment (Supplemental Figure 2F).

Beyond analyzing the effects of the antagonists on the levels of hFcRn-GFP in transfected cells, we also investigated whether the FcRn antagonists affected FcRn levels in cells endogenously expressing the receptor. HULEC-5A, a human lung-derived microvascular endothelial cell line previously utilized for studying hFcRn, was selected (11). Endogenously expressed hFcRn was detected using AF647-labeled Synt002 Fab fragment (Synt002-Fab-AF647). Synt002 is a humanized antibody that has a high binding affinity for hFcRn and competes with albumin for FcRn binding (72, 73). It was therefore necessary to confirm that Synt002-Fab did not compete with the antagonists before using it for detection. In HEK293-hFcRn-GFP cells, colocalization was observed between AF555-labeled IgG1-MST-HN (Supplemental Figure 3A)/HL161BK (Supplemental Figure 3B), hFcRn-GFP, and Synt002-Fab-AF647. Furthermore, to establish that signal from Synt002-Fab-AF647 was a reliable indicator of hFcRn levels, downregulation analyses analogous to those performed in HEK293-hFcRn-GFP cells were carried out. Briefly, at each time point in the downregulation experiment, the cells were fixed, permeabilized, and stained with Synt002-Fab-AF647. The results of the staining (Supplemental Figure 3C) correlated with the GFP signal (Figure 1A), showing that detection with Synt002-Fab-AF647 could be used to indicate hFcRn levels.

Following treatment with FcRn antagonists, HULEC-5A cells were stained with Synt002-Fab-AF647, and levels of AF647 were analyzed using flow cytometry to determine the cellular amount of hFcRn. HULEC-5A cells treated with HL161BK showed a 60% decrease of Synt002-Fab-AF647 signal compared with the medium control group, and no significant difference in signal was observed for the other treatment groups (Figure 1C and Supplemental Figure 3D). A 14% increase in Synt002-Fab-AF647 staining levels was observed in ARGX-113–treated cells following a 24-hour incubation. As in HEK293-hFcRn-GFP and HMEC-1-hFcRn-GFP cells, no significant difference in Synt002-Fab-AF647 signal was observed in HULEC-5A cells treated with IgG1-MST-HN compared with ARGX-113–treated cells (Supplemental Figure 3D).

To study the kinetics of FcRn downregulation in live cells, a 16-hour time course was imaged in HEK293-hFcRn-GFP cells treated with FcRn antagonists (i.e., HL161BK, N027, and ARGX-113), the Fc of a WT IgG1 (Fc-IgG1-WT), or medium only (Figure 1, D and E; Supplemental Figure 4; and Supplemental Videos 1–5). HL161BK treatment led to decreased normalized volume (sum of voxels) for hFcRn-GFP signal over time, with greater than 80% loss of volume for hFcRn-GFP (Figure 1, D and E; Supplemental Figure 4; and Supplemental Video 4). A 35% reduction in normalized volume for hFcRn-GFP was measured upon N027 treatment, while an increase of approximately 10% was observed upon treatment with ARGX-113 compared with untreated or Fc-IgG1-WT–treated cells (Figure 1, D and E; Supplemental Figure 4; and Supplemental Videos 1–3 and 5). These results are therefore consistent with the observations using flow cytometric analyses to assess hFcRn-GFP levels at discrete time points during incubation with the antagonists.

### Differential effects on HSA recycling in HEK293-hFcRn-GFP cells treated with FcRn antagonists.

**Figure 2:**
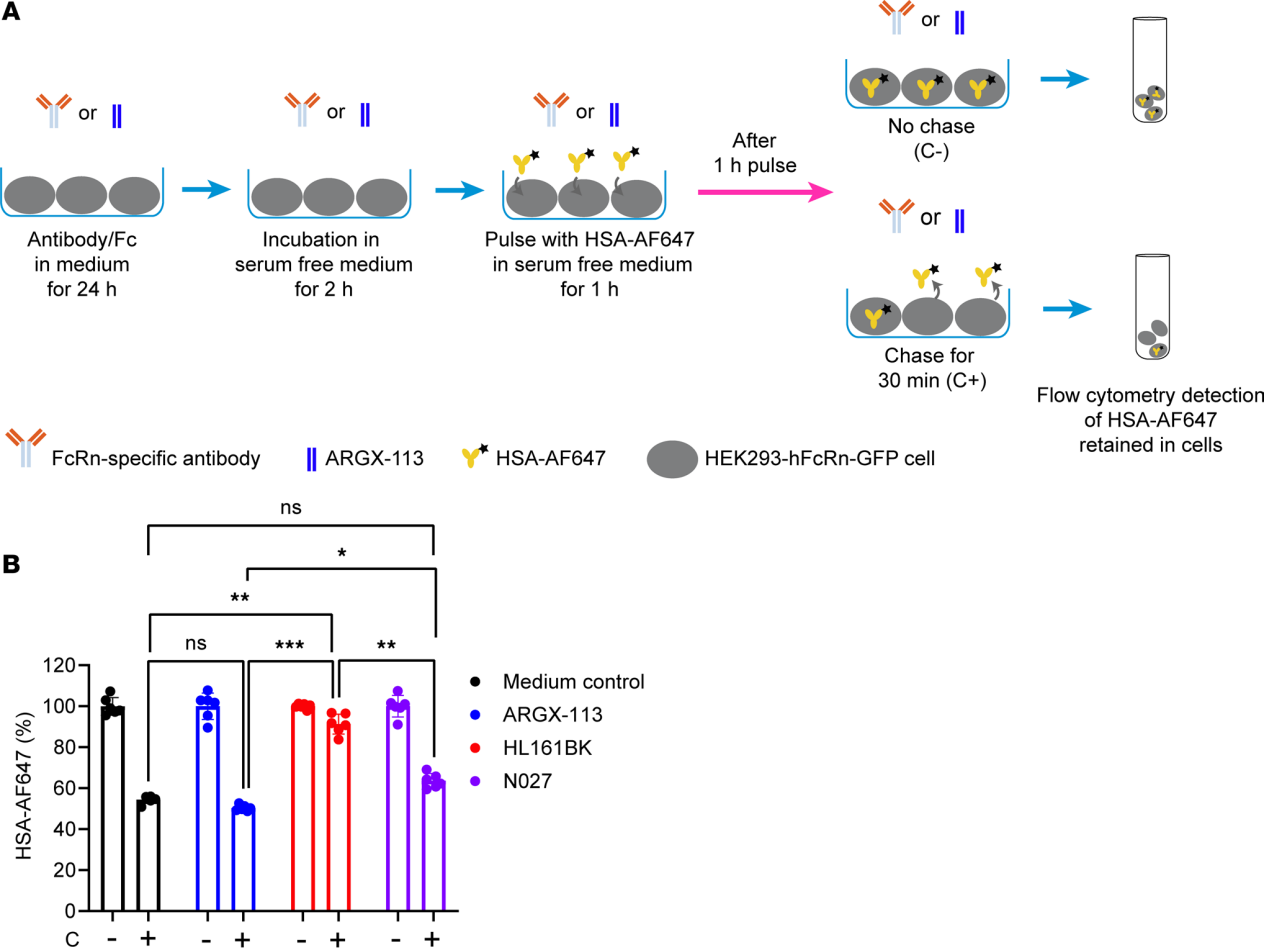
Effects of FcRn antagonists on recycling of HSA by HEK293-hFcRn-GFP cells. HEK293-hFcRn-GFP cells were incubated with 50 nM FcRn antagonist or medium for 24 hours. The cells were then incubated in serum-free medium for 2 hours, pulsed with 250 μg/mL AF647-labeled HSA (HSA-AF647) in serum-free medium for 1 hour, washed, and chased in serum-free medium for 0 (C-) or 30 minutes (C+) at 37°C in a 5% CO_2_ incubator. The cell-associated HSA-AF647 levels following the indicated treatments were determined using flow cytometry. (**A**) Schematic illustration of HSA recycling assay. (**B**) Data normalized against pulse-only levels (represented as 100%). These data are combined from 2 independent experiments, with triplicate samples in each experiment. One-way ANOVA was used for statistical analysis. Statistically significant differences are shown as *P ≤ 0.05, **P ≤ 0.01, ***P ≤ 0.001. Error bars indicate the standard deviation of the mean.

We next assessed the effects of the FcRn antagonists on HSA recycling by HEK293-hFcRn-GFP cells. Cells were incubated with FcRn antagonists and then in serum-free medium. Following this treatment, cells were incubated with AF647-labeled HSA (HSA-AF647) for 1 hour and chased for 0 or 30 minutes (Figure 2A). Cells treated with HL161BK had greater accumulation of HSA-AF647 (pulse-only condition), compared with other groups (Supplemental Figure 5), and showed a substantial reduction in HSA recycling, with similar levels of accumulated HSA-AF647 in cells at the start and end of the chase (Supplemental Figure 5). As a percentage of the HSA present at the end of the pulse, approximately 50% of HSA-AF647 was recycled by cells treated with medium only or ARGX-113; by contrast, less than 10% of HSA-AF647 was recycled in HL161BK-treated cells (Figure 2B). In cells treated with N027, the recycling activity was slightly reduced, with a higher percentage of cell-associated HSA-AF647 following the chase compared with cells treated with ARGX-113 (Figure 2B).

### Binding analyses of competition between FcRn antagonists and albumin for interaction with FcRn.

**Figure 3:**
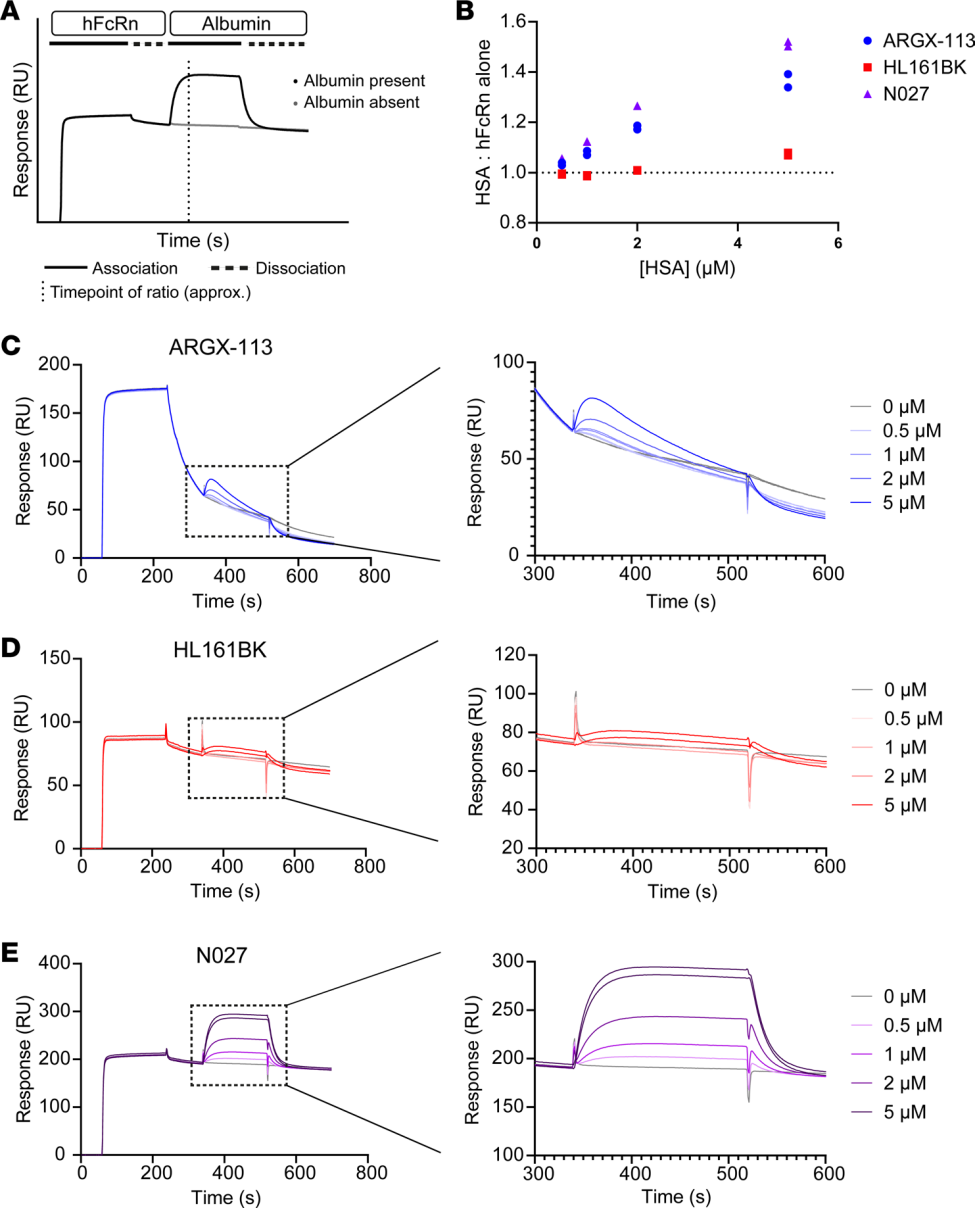
Analyses of competition between FcRn antagonists and HSA for binding to FcRn. hFcRn was injected at a concentration of 350 nM at pH 6.0 across flow cells coupled with FcRn antagonists. Following a brief dissociation period, HSA at concentrations of 0, 0.5, 1, 2, or 5 μM was injected at pH 6.0. The ability of hFcRn to simultaneously bind to HSA and antagonist was evaluated by calculating the maximum ratio of responses (RU) between each HSA injection and PBS-only control. (**A**) Schematic representation of the assay based on the interaction of N027, hFcRn, and albumin. The dotted vertical line approximates the point at which the largest ratios were found. (**B**) Representative data for ratios of signals for HSA/PBS only. Each injection was carried out in duplicate, and the results are representative of 2 independent experiments. Sensorgrams are shown for ARGX-113 (**C**), HL161BK (**D**), and N027 (**E**) in full (left panels) and with the highlighted regions containing the HSA injections expanded (right panels).

Surface plasmon resonance was used to investigate whether hFcRn retains its ability to bind to albumin when in complex with the different FcRn antagonists. Antagonists were covalently coupled to flow cells of a CM5 sensor chip, followed by injection of hFcRn at pH 6.0 and subsequently different concentrations of albumin during the dissociation phase of hFcRn from the antagonist. This approach allowed for the analysis of albumin-hFcRn interactions in the presence of antagonist (Figure 3A). The response for each injection of albumin was divided by the corresponding signal in the matching PBS control time point to produce a single ratio for each concentration of HSA (Figure 3B). hFcRn in complex with both N027 and ARGX-113 can be bound by albumin at concentrations at and above 1 μM. ARGX-113 dissociates relatively rapidly from hFcRn (Figure 3C) (43), particularly in comparison with HL161BK (Figure 3D) and N027 (Figure 3E), which have higher affinities (40, 74) and bind via their Fab arms. As a result, the ratios measured for ARGX-113 could be complicated by the dissociation rate of the corresponding antagonist from hFcRn. In contrast with the observations for N027 and ARGX-113, interaction of hFcRn with HL161BK led to very weak binding of albumin, with signal above background only observed for albumin at a concentration of 5 μM (Figure 3B). The marked reduction in the ability of albumin to bind to FcRn that is captured by HL161BK is consistent with the partial overlap of the binding site of HL161BK and albumin on hFcRn (10, 41, 74).

### Differential lysosomal trafficking behavior of hFcRn and FcRn antagonists in cells transfected with hFcRn-GFP.

**Figure 4:**
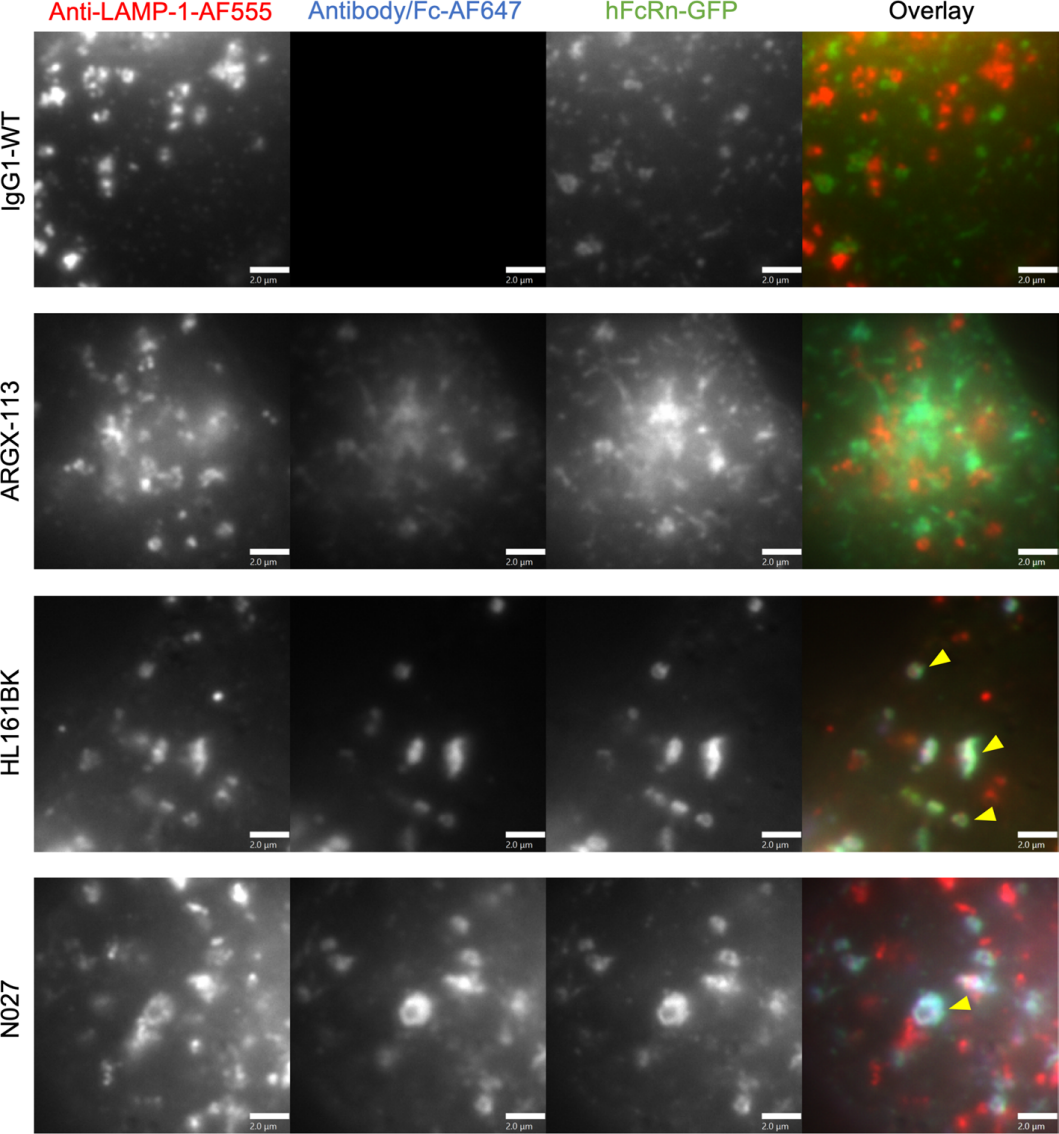
Late endosomal/lysosomal trafficking analysis in HEK293-hFcRn-GFP cells. HEK293-hFcRn-GFP cells were incubated with 50 nM AF647-labeled FcRn antagonist or IgG1-WT (control) for 3 hours. Following incubation, cells were fixed and permeabilized, and detection of late endosomes/lysosomes was carried out using anti–LAMP-1 antibody followed by AF555-labeled goat anti-mouse IgG conjugate. Yellow arrowheads indicate the detection of hFcRn-GFP and AF647 antagonists in anti–LAMP-1–positive compartments. Images for the AF555 channel were adjusted for visualization. Data are representative of 2 independent experiments, each consisting of at least 2 dishes per condition, and at least 6 images for each dish. AF555, AF647, and GFP are pseudocolored red, blue, and green, respectively. Each image represents part of a single cell. Scale bars = 2 μm.

**Figure 5:**
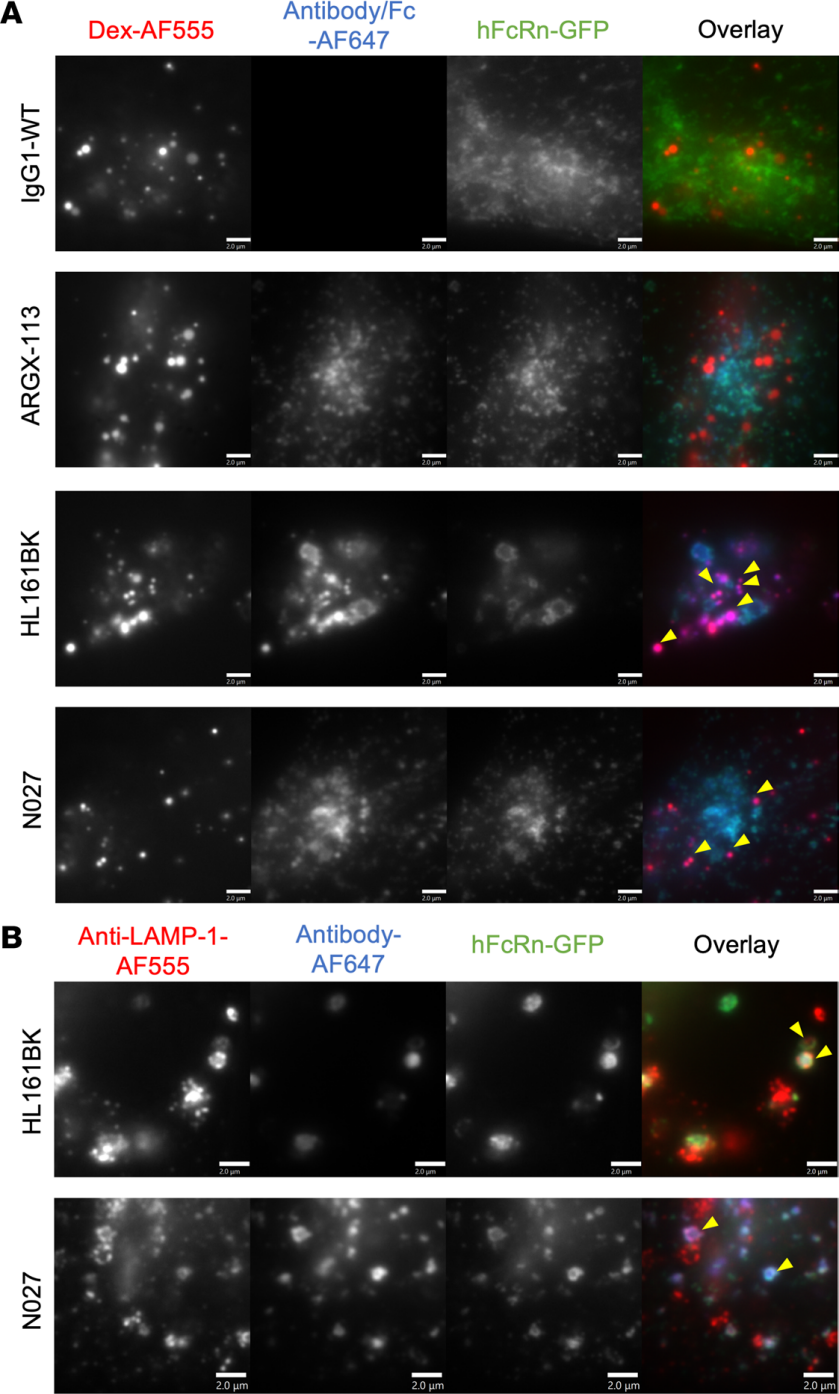
Late endosomal/lysosomal trafficking analyses in HMEC-1-hFcRn-GFP cells. (**A**) HMEC-1-hFcRn-GFP cells were pulsed (1 hour) and chased (6 hours) with 500 μg/mL AF555-labeled dextran (Dex-AF555). Following the 1-hour pulse, cells were incubated with 50 nM AF647-labeled FcRn antagonists or IgG1-WT (control) for 6 hours (i.e., the 6-hour chase of dextran and incubation of FcRn antagonists overlapped). Following incubation, cells were washed, fixed, and imaged. Images were adjusted for the AF555 channel for visualization. Yellow arrowheads indicate the detection of HL161BK/N027-AF647 in dextran-positive compartments. (**B**) HMEC-1-hFcRn-GFP cells were incubated with 50 nM AF647-labeled HL161BK or N027 for 6 hours. Cells were subsequently fixed and permeabilized, and detection of late endosomes/lysosomes was carried out using anti–LAMP-1 antibody followed by AF555-labeled goat anti-mouse IgG conjugate. Yellow arrowheads indicate the detection of HL161BK/N027 and hFcRn-GFP in anti–LAMP-1–positive compartments. Data are representative of 2 independent experiments, each consisting of 2 dishes per condition, and at least 6 images from each dish. AF555, AF647, and GFP are pseudocolored red, blue, and green, respectively. Each image represents part of a single cell. Scale bars = 2 μm.

Since hFcRn levels were differentially affected by the antagonists, we next carried out subcellular trafficking analyses to determine whether there was variation in the delivery of the antagonist and hFcRn to lysosomal compartments within different cells. First, in HEK293-hFcRn-GFP cells, following 3 hours of incubation, HL161BK-AF647 or N027-AF647 and hFcRn-GFP were detectable in LAMP-1^+^ late endosomes/lysosomes, whereas no detectable IgG1-WT-AF647 or ARGX-113-AF647 in these compartments was observed using analogous conditions/parameters for imaging, data processing, and display (Figure 4). Similarly, in HMEC-1-hFcRn-GFP cells, HL161BK-AF647 could be detected in dextran-positive lysosomes following incubation for 6 hours, but GFP fluorescence was not detected in these compartments (Figure 5A). The failure to detect GFP under the conditions of imaging/data processing is most likely due to quenching of GFP fluorescence that occurs at acidic pH (75, 76) in lysosomes. Following 6 hours of incubation, N027-AF647 was detected in dextran-positive lysosomes, whereas under analogous imaging and data-processing conditions, no IgG1-WT-AF647 or ARGX-113-AF647 in lysosomes was detected (Figure 5). Consequently, and consistent with detection of hFcRn-GFP in lysosomes in permeabilized HEK293-hFcRn-GFP cells, we reasoned that permeabilization of HMEC-1-hFcRn-GFP cells in buffer at near-neutral pH may lead to an increase in GFP fluorescence. HMEC-1-hFcRn-GFP cells were therefore incubated with HL161BK-AF647 or N027-AF647 for 6 hours, followed by fixation, permeabilization, and detection of late endosomes/lysosomes using a LAMP-1–specific antibody (Figure 5B). Under these conditions, HL161BK-AF647/N027-AF647 and GFP could be detected in LAMP-1^+^ late endosomes/lysosomes.

### Differential lysosomal trafficking behavior of hFcRn and FcRn antagonists in cells that endogenously express hFcRn.

**Figure 6:**
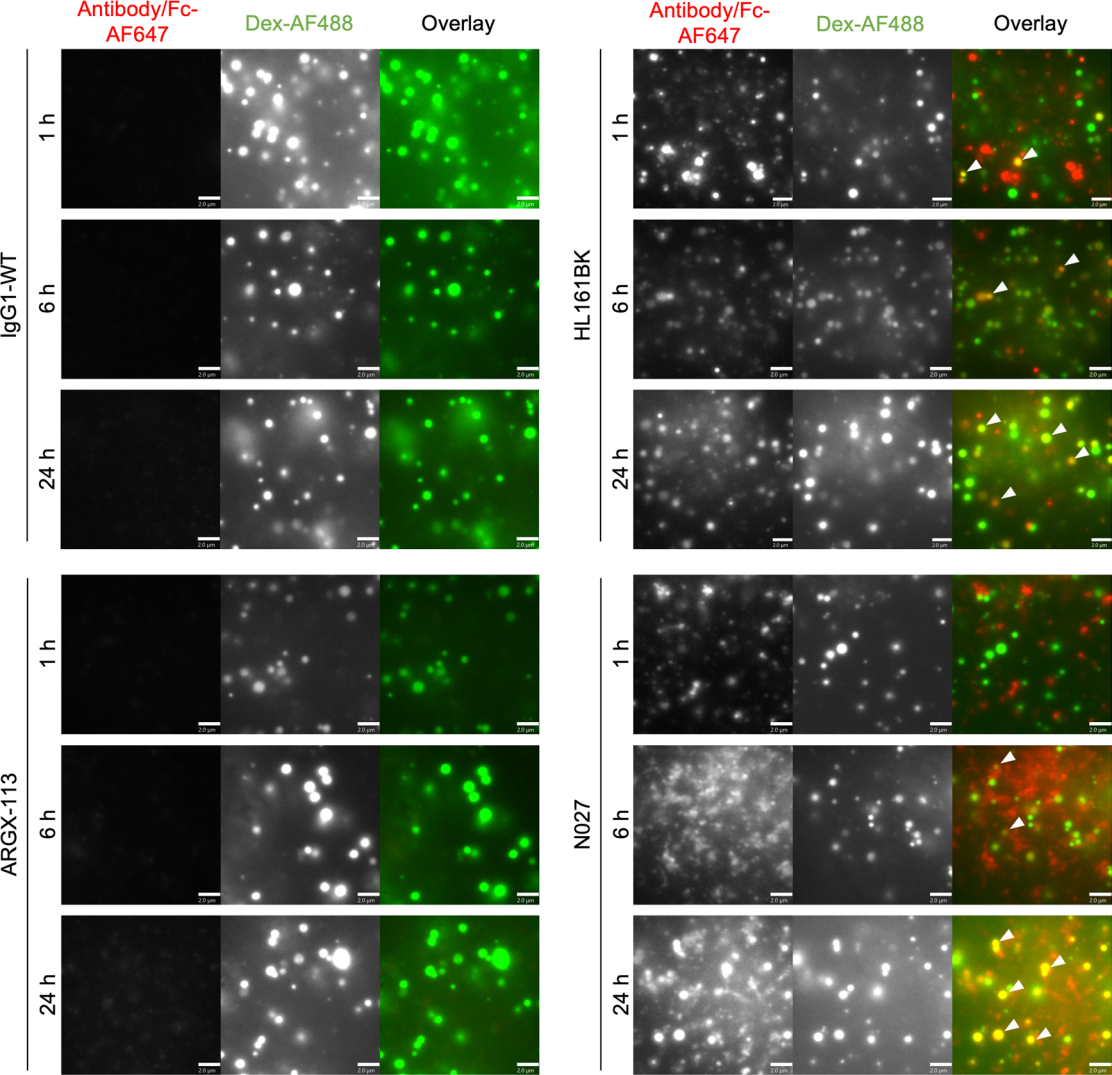
Lysosomal trafficking analyses in HULEC-5A cells. HULEC-5A cells were incubated with 50 nM AF647-labeled FcRn antagonist or an IgG1-WT control for 1, 6, and 24 hours. During these incubations, cells were pulsed (1 hour) and chased (6 hours) with 500 μg/mL Dex-AF488. Following incubation, cells were washed, fixed, and imaged. White arrowheads in the panels indicate the detection of AF647-labeled HL161BK or N027 in dextran-positive compartments. Images were adjusted for the AF488 channel for visualization. Data are representative of 2 independent experiments, each consisting of 2 dishes per condition, and at least 6 images for each dish. AF647 and AF488 are pseudocolored red and green, respectively. Each image represents part of a single cell. Scale bars = 2 μm.

To study the differences in subcellular trafficking in cells that endogenously express human FcRn, HULEC-5A cells were used. Lysosomes were labeled with dextran in the same manner as for HMEC-1-hFcRn-GFP cells, and cells were incubated with labeled FcRn antagonists for 1, 6, and 24 hours. HL161BK-AF647 could be detected in dextran-positive lysosomes in HULEC-5A cells following 1 hour of incubation, and AF647 signal persisted for up to 24 hours of incubation (Figure 6). N027-AF647 was also detected in dextran-positive lysosomes following 6 and 24 hours of incubation. Using analogous imaging and data-processing parameters, ARGX-113-AF647 and IgG1-WT-A647 could not be detected in dextran-positive lysosomes for up to 24 hours of incubation (Figure 6), though, consistent with our previous observations (69), could be detected when the signal was adjusted during processing (Supplemental Figure 6).

### Changes in albumin levels following repeated injections of FcRn antagonists in a humanized mouse model.

**Figure 7:**
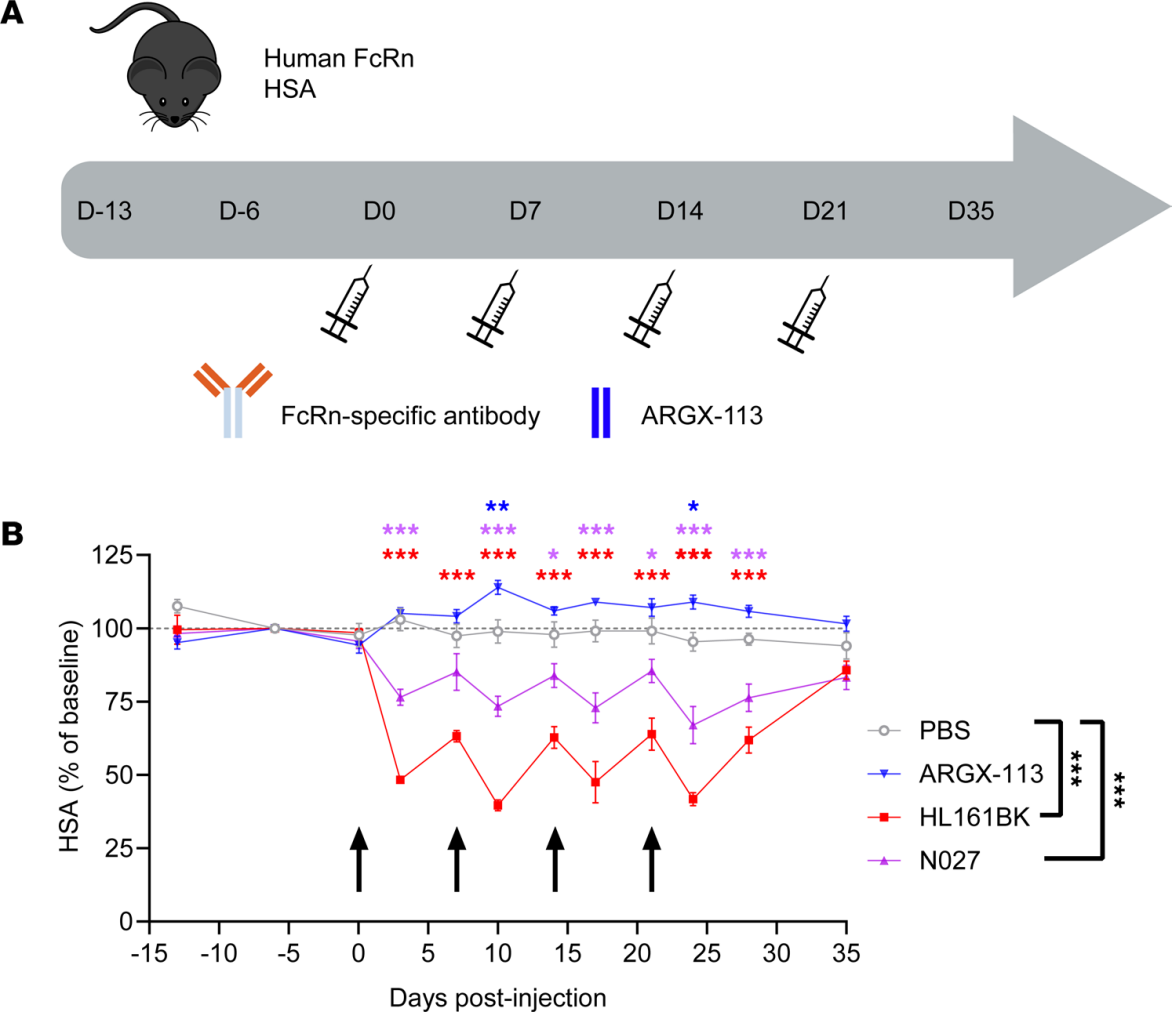
Effects of FcRn antagonists on HSA levels in mice humanized to express hFcRn and HSA. Female or male 14- to 15-week-old Albumus *Rag1*-deficient (KO) mice were IP injected with antagonist (100 mg/kg for N027 or HL161BK, *n* = 5 per treatment group; 35 mg/kg for ARGX-113, *n* = 8) or PBS (*n* = 3) on days 0, 7, 14, and 21. We collected 20 μL blood samples to establish baseline levels of endogenous HSA on days –13 and –6 (predose). We collected 20 μL blood samples from each mouse 1 hour after each injection and samples on days 3, 10, 17, 24, 28, and 35. HSA concentrations were assessed by ELISA. (**A**) Schematic representation of dosing and sample collection. (**B**) HSA levels normalized to day –6; black arrows indicate days of IP injections. Data for PBS, HL161BK, and N027 are representative of 2 individual experiments (*n* = 3 for PBS, *n* = 5 for HL161BK and N027 in each experiment); data for ARGX-113 (*n* = 8) are from 1 experiment. Statistical analysis for each day was performed with a longitudinal model, and significant differences compared with the PBS control are denoted above each time point: *P ≤ 0.05, **P ≤ 0.01, ***P ≤ 0.001. One-way ANOVA with Dunnett’s multiplicity adjustment was used for the analysis of the overall average percentage changes in HSA levels from baseline (PBS control; D0–D35) for the individual mouse profiles over time, summarized as AUC (significant differences denoted on the right of the key). Error bars indicate the standard error of the mean.

To extend our observations to an in vivo model, the effects of the FcRn antagonists on albumin homeostasis were evaluated using a humanized mouse model [C57BL/6N-*Fcgrt*^tm1.1(huFCGRT)Geno^ *Alb*^tm1.1(huALB)Geno^ *Rag1*^tm1Geno^; Albumus *Rag1*-deficient mice]. The mice used expressed both hFcRn and HSA but lacked B cells, T cells, and endogenous immunoglobulin due to KO of the *Rag1* gene (77). Four doses of HL161BK, N027, or ARGX-113 were administered to the animals once weekly via intraperitoneal (IP) delivery (Figure 7A). The concentrations of HSA were significantly reduced 72 hours after administration of both full-length antibody antagonists, and this effect was observed following repeat dosing of each antagonist. Specifically, HL161BK delivery resulted in a significant decline in albumin levels in all samples collected between days 3 and 28, while N027 produced a decline for all time points except day 7 (*P* = 0.058) (Figure 7B). Consistent with the in vitro cellular analyses and observations in clinical trials, the largest effect (evaluated as the AUC compared with the PBS control, day 0 to day 35; D0–D35) was observed for HL161BK, where albumin levels declined at each injection cycle to a nadir of approximately 50% (Figure 7B). Consistent with prior observations, the reduction following N027 administration was more modest, but the AUC was also significantly different from baseline. Finally, the ability of the in vivo model to recapitulate clinical observations (50) extended to ARGX-113, which led to a significant increase in albumin levels relative to PBS on D10 and D24. However, this modest increase in albumin following ARGX-113 administration was not significant when the AUC was analyzed (*P* = 0.083). Serum albumin levels returned to original concentrations within 2 weeks following the final dose of each FcRn antagonist.

## Discussion

Targeting of the FcRn to reduce IgG concentrations became a clinically validated strategy with the 2021 approval of efgartigimod for treatment of generalized, acetylcholine receptor antibody–positive myasthenia gravis. Efgartigimod (ARGX-113) is an engineered Fc fragment containing the Abdeg mutations that increase affinity for FcRn in both acidified endosomes and at near-neutral extracellular pH (43). The engineering of the natural FcRn interaction site for increased affinity results in retention of pH dependence of efgartigimod for FcRn binding, with a substantially higher affinity at acidic pH than near-neutral pH (43). This property allows efgartigimod to outcompete endogenous IgG for FcRn binding, resulting in enhanced degradation of the latter following in vivo delivery (45, 69), while efgartigimod itself retains some recycling activity (69). Other therapeutic candidates that inhibit FcRn-mediated recycling of IgG differ from efgartigimod insofar as they are full-length antibodies that block the binding site for IgG (Fc) on FcRn via high-affinity interactions of their Fab arms (38, 40, 78). The combination of variable domain-FcRn interactions and the conventional, lower affinity FcRn binding sites located in the CH2-CH3 domains of the Fc fragment results in 4 potential binding sites for FcRn on the full-length antibody class of FcRn antagonists. To date, reduced serum albumin levels have been reported during clinical trials using 2 of the candidates in the full-length antibody class (40, 49). In the current study, we explore underlying cellular and molecular mechanisms that might contribute to these effects on albumin.

Albumin plays diverse roles in the body that include transport of free fatty acids, bilirubin, amino acids, and albumin binding drugs (51–54). Consequently, abnormally low serum albumin levels can lead to increased risk of cardiovascular disease and toxicity of drugs that have narrow therapeutic indices (55–60). Given the importance of maintaining albumin levels within the normal range in the body, it is of relevance to investigate the mechanisms by which FcRn antagonists might modulate the concentrations of this abundant serum protein. The current study shows that albumin-lowering effects of full-length antibody antagonists (HL161BK and N027) can reduce albumin recycling relative to efgartigimod (ARGX-113) in an in vitro cell-based assay (Figure 2B) and identifies 2 possible mechanisms through which these molecules can lead to decreased levels of circulating albumin. These 2 antagonists bind to FcRn with high affinity in the pH range 6.0–7.4, and although both inhibit the binding of IgG to FcRn, they interact with distinct epitopes of FcRn (41, 42). Coincubation of HL161BK with several hFcRn-expressing cell lines, some expressing hFcRn endogenously and others as the result of transfection with constructs encoding hFcRn-GFP, led to reductions in hFcRn or hFcRn-GFP levels within 1 hour of antagonist exposure. Consistent with the rapid loss of hFcRn in the presence of HL161BK, microscopy analyses demonstrated the delivery of hFcRn-GFP and the antagonist to late endosomes or lysosomes within several hours of antagonist treatment. In contrast with HL161BK, incubation of hFcRn- and hFcRn-GFP–expressing cells with N027 had variable effects on FcRn levels in cells: in stably transfected HEK293 cells that overexpress hFcRn-GFP, a 30% reduction in hFcRn-GFP levels was observed, whereas in other cell types (hFcRn-GFP–transfected HMEC-1 cells or HULEC-5A cells endogenously expressing hFcRn), there were no significant reductions. Despite these different effects on levels of hFcRn(-GFP) following N027 treatment, in both transfected cell lines, hFcRn(-GFP) could be detected in late endosomes or lysosomes at higher levels compared with that observed in cells treated with ARGX-113. In this context, the reduction of hFcRn(-GFP) levels for HL161BK-treated cells was also lower in transiently transfected HMEC-1 cells and (untransfected) HULEC-5A cells than in transfected HEK293 cells, suggesting that there may be compensatory mechanisms for accelerated degradation of hFcRn in some cell types. In addition to the effects of HL161BK and N027 on the subcellular trafficking behavior of hFcRn, a second mechanism for albumin reduction, involving blockade of binding of albumin to hFcRn, was observed only for HL161BK. This inhibition is consistent with the partial overlap of the binding site on hFcRn with the residues involved in albumin binding (41), whereas N027 binds to a distinct site (10, 42).

Interestingly, the incubation of hFcRn-expressing cells with ARGX-113 led to slight elevations in hFcRn and hFcRn-GFP levels. This is consistent with observations in clinical trials using efgartigimod (50), indicating increased albumin concentrations that remain within the normal range can occur during treatment with this therapeutic.

Our observations raise questions concerning the molecular nature of the differential effects of the FcRn antagonists on albumin homeostasis. Aside from the competition of HL161BK with albumin for FcRn binding, both HL161BK and N027 with 4 potential binding sites for FcRn lead to increases in FcRn degradation in cells and reduced albumin recycling, whereas efgartigimod (ARGX-113) with 2 Fc-based binding sites does not. In a cellular model, multivalent immune complexes consisting of antigen bound to multiple IgG molecules were shown to induce cross-linking of HA-tagged FcRn, thereby diverting this receptor into lysosomes, whereas complexes comprising engineered IgG molecules that do not interact with FcRn lacked this activity (79). In the same setup, an anti-HA antibody cross-linked by an anti-IgG antibody, but not monomeric IgG, also drove the transport of FcRn into lysosomes, thereby indicating the necessity of larger immune complexes for FcRn clustering. The rescue of FcRn-bound ligands from lysosomal degradation within cells involves endosomal sorting into tubulovesicular carriers (TCs) (12, 79, 80). Regulators of cellular trafficking such as Rab GTPases, in combination with motor proteins, play a critical role in driving TC formation (81, 82). The narrow dimensions of endosomally derived tubules have been reported to exclude large, FcRn-bound molecular aggregates such as multivalent immune complexes (79). Sorting of membrane receptors into the recycling pathway has been proposed to occur either by geometry-based sorting because of the high surface area/volume ratio of tubules or by interactions of cytosolic tail motifs of membrane receptors with specific sorting proteins (83). Consequently, different orientations and epitopes of binding of FcRn antagonists may result in configurations and/or valencies of membrane-associated FcRn that are incompatible with endosomal sorting into the recycling pathway but instead lead to lysosomal delivery.

One process leading to lysosomal delivery, autophagy, can be starvation induced (84–86). Autophagy has been shown to regulate FcRn expression levels and recycling activity in renal tubule epithelial cells and macrophages (84, 87). However, for several reasons, it is unlikely that the data presented in the current manuscript showing that HL161BK leads to substantial reductions in FcRn expression levels, or FcRn-mediated recycling of albumin, are due to starvation-induced autophagy. First, our flow cytometry experiments to assess effects of antagonists on FcRn(-GFP) levels were carried out using complete medium, not under conditions of nutrient deprivation that have been reported to lead to autophagy upregulation (84). Second, although cells were nutrient deprived for up to 2 hours before analyses of effects of antagonists on IgG or albumin recycling, the treatment of cells with different antagonists or controls was performed under analogous culture conditions.

An important outcome of this study is that we show that the delivery of HL161BK and N027 into mice humanized for FcRn and albumin leads to decreased albumin levels, which are reversible after treatment cessation. In addition, ARGX-113 delivery led to reversible, moderate increases in albumin levels, consistent with clinical observations where efgartigimod can result in higher albumin concentrations that fall within the normal range (50). These results indicate that this model can be used to recapitulate the observations in patients.

In summary, our studies provide mechanistic insight into possible pathways by which full-length FcRn-specific antibodies can lead to reductions in albumin levels. Diversion of FcRn into degradative lysosomes can contribute to lower albumin recycling activity, and for HL161BK, direct competition for albumin binding limits receptor availability. Collectively, these analyses not only reveal insight into clinical observations using FcRn antagonists but also have important implications for design principles for this emerging class of therapeutics.

## Methods

### Sex as a biological variable.

Male and female mice were utilized. Sex was not considered as a biological variable.

### Methods available as supplemental material.

The methods describing size-exclusion chromatography, IgG recycling assays, hFcRn detection using a fluorescently labeled Fab fragment, and immunoblotting of hFcRn are described in Supplemental Methods.

### Recombinant protein production.

The following antibodies or Fc fragments were expressed and purified for use in the current study: 2 full-length human IgG1 antibodies using the sequences of HL161BK (41) and N027 (42) (i.e., analogs of batoclimab and nipocalimab, respectively); a WT Fc fragment (Fc-IgG1-WT) and an Fc fragment containing the Abdeg mutations (ARGX-113, an efgartigimod analog) (68, 69); and a full-length IgG1 that binds to hen egg lysozyme with a WT Fc sequence (IgG1-WT) or the Abdeg mutations (IgG1-MST-HN) (70) (Supplemental Figure 1A). Expression and purification of Fc-IgG1-WT, HL161BK, and N027 were carried out at Evitria SA as described previously (69). ARGX-113 (88) was produced by Lonza Biologics using a CHOK1SV GS-KO cell line (Lonza Group Ltd.), as described previously (69). Anti-hen egg lysozyme recombinant human IgG1 proteins (IgG1-WT and IgG1-MST-HN) were purified from culture supernatants using lysozyme-sepharose as described (43, 70). The Fab fragment of Synt002 (72, 73) was expressed and purified at Evitria SA.

The soluble, extracellular domains of the hFcRn were produced by cotransfection of HEK293 cells with pcDNA3.4 constructs encoding β2m (UniProt ID P61769) and the FcRn-α heavy chain (UniProt ID P55899, residues 24–297) appended with a C-terminal hexahistidine tag. The α-chain construct was generated using the methods and plasmids originally used for expression in insect cells described in earlier work (89). Recombinant, histidine-tagged hFcRn was purified using Ni^2+^-NTA agarose (QIAGEN), and aggregates were removed using size-exclusion chromatography with a HiLoad 16/600 Superdex 200 pg column (Cytiva).

Plasmids used for expression of hFcRn with C-terminally fused enhanced GFP and human β2m have been described (12).

### Labeling of proteins.

Proteins (hIgG, HSA, Synt002-Fab, IgG1-WT, and all FcRn antagonists) in Dulbecco’s phosphate-buffered saline (DPBS; Lonza, 17-512Q) at 2 mg/mL were buffer-exchanged into 100 mM NaHCO_3_ (pH 8.3) with Zeba Spin Desalting Columns (Thermo Fisher Scientific, 89893) according to the manufacturer’s instructions. AF647 dyes (Invitrogen, A37573) were prepared at a concentration of 20 μg/μL in anhydrous DMSO (Invitrogen, 2025920). To achieve a degree of labeling of 2 dye molecules per protein molecule, a 2-fold molar excess of AF647 dye was added to buffer-exchanged protein and incubated for 1 hour in the dark at room temperature. Then the mixture was centrifuged at 9,500*g* at 4°C for 15 minutes. The supernatant was dialyzed against DPBS for 3 days in the dark at 4°C using a dialysis membrane (6–8 kD MWCO, Spectra/Por 1, 3312928). Following dialysis, if size-exclusion analyses indicated greater than 1% aggregates, labeled proteins were purified using an NGC Quest 10 Chromatography System (Bio-Rad) and a HiLoad 16/600 Superdex 200 pg column. The labeled proteins were filtered through 0.22 μm syringe filters (Olympus, 25–243) and quantified using a NanoDrop One (Thermo Fisher Scientific).

### Cell culture.

HEK293 cells (CLS, 300192) were stably transfected with expression plasmids encoding hFcRn-GFP and β2m (HEK293-hFcRn-GFP) as described previously (68) and maintained in DMEM (Corning, 10-017-CM) with 10% FBS (Gibco, 10270-106), 1% penicillin-streptomycin (Gibco, 15140122), 1% GlutaMAX (Gibco, 35050), and 1% sodium pyruvate (Gibco, 11360). HMEC-1 (12, 71) and HULEC-5A (11) cells (available from ATCC; CRL-3243 and CRL-3244, respectively) were maintained in MCDB-131 (Gibco, 10372019) with 10% FBS, 1% penicillin-streptomycin, and 1% l-glutamine (Gibco, 25030024). HMEC-1 cells were transiently transfected with expression plasmids encoding hFcRn-GFP and β2m using a Nucleofector 2b device (Amaxa) as described (12). Unless specified, all media used were pH 7.4.

### Flow cytometric analyses of FcRn levels.

HEK293-hFcRn-GFP cells were seeded at 75,000 cells/well in phenol red–free DMEM (pH 7.4 unless specified otherwise) with 10% FBS (bovine IgG depleted) overnight in 24-well plates (Corning, 3524). Then cells were incubated with 50 nM FcRn antagonist or medium alone for 1, 3, 6, 12, or 24 hours at 37°C in a 5% CO_2_ incubator. Cells were washed with ice-cold DPBS at the end of the incubations, trypsinized, washed with ice-cold DPBS, and fixed with 3.4% paraformaldehyde (PFA) for 15 minutes at room temperature. Cells were washed with ice-cold DPBS and stored in DPBS + 1% BSA, and the GFP fluorescence signal was acquired using a CytoFLEX S flow cytometer (Beckman Coulter).

To investigate how the concentration of HL161BK and N027 affects FcRn downregulation in HEK293-hFcRn-GFP cells, 75,000 cells/well were incubated with 5, 50, and 500 nM HL161BK and N027 for 1, 12, or 24 hours at 37°C in a 5% CO_2_ incubator. Then cells were treated as described above, and the GFP fluorescence signal was acquired using a CytoFLEX S flow cytometer.

For analyses of FcRn downregulation in the presence of HSA and hIgG in HEK293-hFcRn-GFP cells, similar conditions were used to those described above, but cells were incubated with 50 nM FcRn antagonist together with 6 μM HSA (MilliporeSigma, A3782, purified using size-exclusion chromatography prior to use) and 2 μM hIgG. For controls, both medium only and medium containing 6 μM HSA and 2 μM hIgG were included.

Transiently transfected HMEC-1 cells (12) were resuspended in phenol red–free Ham’s F12K (US Biologicals, D9811-14C, pH 7.4 unless specified otherwise) with 10% FBS (bovine IgG depleted) and seeded at 75,000 cells/well into 24-well plates overnight. Subsequently, cells were incubated with 50 nM FcRn antagonist or medium only for 1, 3, 6, 12, or 24 hours at 37°C in a 5% CO_2_ incubator. Addition of antagonists was carried out with staggering so that all samples were harvested at the same time following incubations. Cells were washed with ice-cold DPBS at the end of incubations, trypsinized, washed, and fixed, and data were acquired as for HEK293-hFcRn-GFP cells. hFcRn-GFP–positive populations (determined by using untransfected cells) were gated for the analysis, and a fixed gating strategy was applied in FlowJo for data analysis.

HULEC-5A cells were seeded into 24-well plates overnight at 75,000 cells/well in the same medium as used for HMEC-1 cells. Cells were incubated with 50 nM FcRn antagonist or medium only for 1, 3, 6, 12, or 24 hours at 37°C with 5% CO_2_. Cells were washed with ice-cold DPBS, trypsinized, washed with DPBS, and fixed with 3.4% PFA for 15 minutes at room temperature. The cells were permeabilized with 0.25 mg/mL saponin in DPBS for 20 minutes at room temperature followed by washing with DPBS and blocking with 4% BSA for 30 minutes at room temperature. Subsequently, the cells were washed with DPBS and incubated with 2 μg/mL Synt002-Fab-AF647 in DPBS + 1% BSA with 0.25 mg/mL saponin for 30 minutes at room temperature. Finally, cells were washed with DPBS and resuspended in DPBS + 1% BSA, and data were acquired using a CytoFLEX S flow cytometer.

### Live-cell imaging of HEK293-hFcRn-GFP cells.

HEK293-hFcRn-GFP cells were seeded onto poly-l-lysine–coated (MilliporeSigma, P4707) μ-slide 8-well, ibiTreat, tissue culture–treated polymer coverslips (Ibidi, 80826) overnight at 20,000 cells/well in growth medium. The following day, cells were washed with prewarmed Fluorobrite imaging medium (Thermo Fisher Scientific, A1896701, pH 7.4 unless specified otherwise) complemented with penicillin-streptomycin, l-glutamine (Merck Life Science BV, G7513-100ML), and 1% BSA (Merck Life Science BV, A7906) and incubated for 1 hour at 37°C with 5% CO_2_. Immediately before imaging, FcRn antagonists were added to the medium, which is defined as *t_0_*. Three *x,y* positions per condition were selected for time-lapse recording using a confocal spinning disk system (ZEISS). This system includes an Observer Z.1 microscope equipped with a Yokogawa disk CSU-X1. *Z*-stacks (interval: 0.57 μm) of the GFP signal were taken with a plan Apo 40×/1.4 oil DIC III objective and a Photometrics Prime 95B camera every 20 minutes over 16 hours. During imaging, the cells were maintained in 5% CO_2_ at 37°C. The focus was stabilized over time by the Definite Focus 2.0 (ZEISS).

After acquisition, a pixel reassignment algorithm in combination with a Wiener filter was carried out before analysis.

The processed 4D data sets were analyzed using Volocity 6.3 (Quorum Technologies). A voxel-based intensity threshold analysis of ±15 cells per condition was carried out, and the corresponding voxel counts were exported to GraphPad Prism 7. Graphs of the volume (sum of voxels) were plotted over time for the GFP signal. The resulting values were normalized to *t_0_*, which was defined as 100%.

The time-lapse images were processed into movies using Fiji (ImageJ, NIH), in which the maximum intensity projection was used to display the *Z*-stacks of the fluorescence signals at each time point.

### Analyses of HSA recycling by HEK293-hFcRn-GFP cells.

HEK293-hFcRn-GFP cells were seeded at 75,000 cells/well in phenol red–free DMEM (pH 7.4 unless specified otherwise) with 10% FBS (bovine IgG depleted) in 24-well plates overnight. Subsequently, the cells were incubated with 50 nM FcRn antagonist (pH 7.4 unless specified otherwise) for 24 hours at 37°C in a 5% CO_2_ incubator. Following incubation for 2 hours in FBS-free DMEM (phenol red–free, containing 50 nM FcRn antagonist) at 37°C in 5% CO_2_, cells were pulsed with 250 μg/mL HSA-AF647 for 1 hour in FBS-free DMEM (phenol red–free, containing 50 nM FcRn antagonist) followed by washing with DPBS at room temperature and chasing for 30 minutes in FBS-free DMEM (phenol red–free, containing 50 nM FcRn antagonist). This pulse was followed by washing with ice-cold DPBS (pulse only; no chase) or washing with DPBS at room temperature and chasing for 30 minutes in FBS-free DMEM (phenol red–free, containing 50 nM FcRn antagonist). At the end of the 30-minute chase period, cells were washed with ice-cold DPBS, then trypsinized, washed with DPBS, and fixed with 3.4% PFA for 15 minutes at room temperature. Data were acquired using a CytoFLEX S flow cytometer.

### Surface plasmon resonance analyses.

Competition between albumin and FcRn antagonist for binding to hFcRn was assessed using surface plasmon resonance on a BIAcore T200 (Cytiva). FcRn antagonists were immobilized using amine coupling chemistry at approximately 300–650 response units on flow cells of a CM5 sensor chip (Cytiva), and 350 nM hFcRn was injected over the flow cells at 10 μL/min in DPBS + 0.01% (v/v) Tween 20 + 0.05% sodium azide (Severn Biotech Ltd, 40-2010-01) (pH 6.0) (assay buffer). This was followed by injections of HSA (MilliporeSigma, A3782) using a flow rate of 10 μL/min at concentrations ranging 0.5–5 μM in pH 6.0 assay buffer or assay buffer alone. At the end of each cycle, the flow cells were regenerated using 50 mM sodium phosphate (pH 12.0) (Thermo Fisher Scientific, 10345720 and 10684732).

The results of the assay were reported as the largest ratio between the response when albumin was present and the corresponding signal from the matched buffer-only injection. Ratios were computed using R v. 4.2.0 (R Project for Statistical Computing) and RStudio v. 2022.07.0 (Posit) and the results visualized using GraphPad Prism 9 (Dotmatics).

### Lysosomal trafficking analyses in cells transfected with hFcRn-GFP.

HEK293-hFcRn-GFP cells were seeded onto poly-l-lysine–coated MatTek dishes (P35-10-C-NON) fitted with cover glasses (Electron Microscopy Sciences, no. 1.5, 22 mm diameter, 72224-01) overnight at 20,000 cells/well in phenol red–free DMEM with 10% FBS (bovine IgG depleted). The following day, 50 nM AF647-labeled FcRn antagonists were added and incubated for 3 hours at 37°C in a 5% CO_2_ incubator. Subsequently, cells were washed with ice-cold DPBS and fixed with 3.4% PFA at room temperature for 15 minutes. Cells were then washed with DPBS and permeabilized with 0.25 mg/mL saponin in DPBS for 20 minutes at room temperature, followed by washing and blocking with 4% BSA for 30 minutes at room temperature. Cells were then incubated with 10 μg/mL anti–LAMP-1 antibody (Developmental Studies Hybridoma Bank, mouse IgG1, clone H4A3) (12) in DPBS + 1% BSA for 30 minutes at room temperature, followed by washing and blocking with 1% goat serum (MilliporeSigma, G6767) in DPBS + 1% BSA for 30 minutes at room temperature. Cells were washed and incubated with 4 μg/mL AF555-labeled goat anti-mouse IgG conjugate (Invitrogen, A21424) in DPBS + 1% BSA with 0.25 mg/mL saponin for 30 minutes at room temperature. Finally, cells were washed and stored in DPBS + 1% BSA for imaging.

HMEC-1-hFcRn-GFP cells were resuspended in phenol red–free Ham’s F12K with 10% FBS (bovine IgG depleted) and seeded at 10,000 cells/dish in MatTek dishes fitted with cover glasses overnight. Cells were pulsed for 1 hour with 500 μg/mL Dex-AF555 (Invitrogen, D34679), then washed followed by replacement of medium with phenol red–free Ham’s F12K with 10% FBS (bovine IgG depleted) and incubation for a further 6 hours (chase of Dex-AF555) at 37°C in a 5% CO_2_ incubator. During the 6-hour chase period, 50 nM AF647-labeled FcRn antagonists were added. The cells were then washed with ice-cold DPBS and fixed with 3.4% PFA at room temperature for 15 minutes. Following fixation, cells were washed and stored in DPBS + 1% BSA for imaging.

For staining of HMEC-1-hFcRn-GFP cells with anti–LAMP-1 antibody, cells were transfected as described above, then incubated with AF647-labeled HL161BK/N027 for 6 hours. Subsequently, cells were fixed, permeabilized, and stained as described for HEK293-hFcRn-GFP cells.

### Lysosomal trafficking analyses in HULEC-5A cells.

HULEC-5A cells were seeded in MatTek dishes as described above for HMEC-1 cells. Cells were pulsed for 1 hour with 500 μg/mL Dex-AF488, then washed with DPBS at room temperature and chased for 6 hours. Following the chase period, cells were incubated with 50 nM AF647-labeled FcRn antagonists in phenol red–free Ham’s F12K with 10% FBS (bovine IgG depleted), respectively, for 1, 6, and 24 hours at 37°C in a 5% CO_2_ incubator. For example, for 24 hours of incubation, cells were first incubated with AF647-labeled FcRn antagonists for 17 hours, then pulsed for 1 hour with 500 μg/mL Dex-AF488 (in the presence of AF647-labeled FcRn antagonists), and then chased for 6 hours (in the presence of AF647-labeled FcRn antagonist). Following the chase periods, cells were washed with ice-cold DPBS, fixed, and stored in DPBS + 1% BSA at 4°C before imaging.

Cells were imaged using a ZEISS (Axio Observer Z1) inverted epifluorescence microscope equipped with a 100× Plan-APOCHROMAT objective (1.4 NA, 440780-9904), a 1.0× optovar, and an ORCA-Flash 4.0 V3 Digital CMOS camera (Hammamatsu). A broadband LED lamp (X-Cite Xylis XT720S, Excelitas Technologies) was used as the excitation source, and fluorescence filter sets for GFP (excitation: FF01-466/40, dichroic: FF495-Di03, emission: FF01-525/50), AF555 (excitation: FF01-543/22, dichroic: FF562-Di03, emission: FF01-593/40), and AF647 (excitation: FF01-628/40, dichroic: FF660-Di02, emission: FF01-692/40) were used to acquire images. All filter sets were purchased from Semrock.

Identical microscopy settings were used for each fluorophore during imaging (including exposure times). All data were processed and displayed using Lumio (Astero Technologies). Images were piecewise linearly adjusted and cropped for display purposes. Unless indicated otherwise (figure legends), identical processing and display settings were used for each fluorophore.

### Studies in mice expressing hFcRn and HSA.

Approximately 14- to 15-week-old female or male Albumus *Rag1*-deficient (KO) mice [C57BL/6N-*Fcgrt*^tm1.1(huFCGRT)Geno^ *Alb*^tm1.1(huALB)Geno^ *Rag1*^tm1Geno^ purchased from GenOway] (77), humanized for expression of both hFcRn and HSA, were used for the animal studies. On days –13 and –6 (predose), 20 μL blood samples were collected to establish baseline levels of endogenous HSA. On days 0, 7, 14, and 21, the test articles (100 mg/kg for N027 or HL161BK, *n* = 5 per treatment group; 35 mg/kg for ARGX-113, *n* = 8) or PBS (*n* = 3 mice per treatment group) were administered intraperitoneally. We collected 20 μL blood samples from each mouse 1 hour after each injection and on days 3, 10, 17, 24, 28, and 35. After blood collection, Microvette tubes (Sarstedt, 20.1290) were incubated for 30 minutes at room temperature to allow for coagulation. Tubes were then centrifuged for 5 minutes at 5,000*g* at 4°C to separate blood from serum. Samples were stored at –80°C in a 96-well plate (Falcon 96-well Clear V-Bottom Not Treated Polypropylene Storage Microplate). HSA concentrations in serum samples were assessed by a direct sandwich ELISA.

### Quantitation of HSA in serum samples using ELISA.

We coated 96-well microplates (Nunc, 442404) with 1 μg/mL goat anti-human albumin antibody (MilliporeSigma, A1151) in 1× PBS, pH 7.4, before sealing plates and incubating overnight at 4°C. The plates were then washed 3 times with pH 7.4 PBS/0.05% Tween-20, before BLOK casein in PBS (G-Biosciences, 786–194) was added and incubated for 2 hours at room temperature with shaking at 450 rpm. After incubation, the plates were washed as above. Diluted study samples and standards (0–500 ng/mL of HSA; MilliporeSigma, A3782) were added to the plates and incubated for 1 hour with 450 rpm shaking. The plates were washed 5 times as described above, before the detection antibody, goat anti-human albumin (polyclonal antigen affinity purified) conjugated to HRP (Bethyl, A80-129P), at a 1:32,000 dilution in 1× PBS pH 7.4, was added to all wells and the plate incubated for 1 hour with shaking. Last, TMB substrate (CL07, MilliporeSigma) was equilibrated to room temperature and added to all wells after washing 5 times. After 20 minutes, the reaction was stopped by addition of 0.5 M H_2_SO_4_ (Chem-Lab, CL05.2615), and absorbance was read immediately at 450 nm on a TECAN Sunrise plate reader.

### Statistics.

The statistical analysis for FcRn downregulation data was performed by linear mixed models with a Hommel multiplicity correction for the log-transformed MFIs normalized to untreated as a dependent variable. Time, treatment group, and their interaction were included as independent fixed categorical effects and experiment as a random effect. For HMEC-1-hFcRn-GFP and HEK293-hFcRn-GFP cells, different residual variabilities per time point were included, while for HULEC-5A cells, a random effect of the interaction between experiment and treatment group was included in the model to satisfy the assumptions of normality and homoscedasticity of the residuals. One-way ANOVA with Tukey multiplicity correction was applied for analysis of log-transformed HSA recycling data normalized to the untreated condition and for the HSA recycling data normalized to the pulse of individual treatments. A linear mixed model with a first-order auto-regressive correlation structure was selected to analyze the data of the in vivo study applying treatment, time, and their interaction as fixed effects, including exploratory post hoc Dunnett’s multiplicity correction per time point for comparisons with the PBS group. The overall average percentage changes from baseline in HSA levels over time (D0–D35), summarized as AUC, were analyzed using 1-way ANOVA with Dunnett’s multiplicity adjustment. The latter analysis was also applied to the IgG recycling data normalized to the pulse of individual treatments. All statistical models satisfied the normality and homoscedasticity assumptions of the residuals, which were visually checked with a normal quantile-quantile plot and residual versus prediction plot, respectively. All hypothesis tests were performed at a 5% significance level, and multiplicity corrections were applied to control the overall type I error rate at 5%. *P* < 0.05 was considered significant. Statistical analyses were performed with SAS Life Science Analytics Framework version 5.4 and R version 4.2.2.

### Study approval.

The study in mice was performed at the animal facility of VIB, Center for Inflammation Research. The protocol for this experiment was approved by the animal ethics committee at VIB with EC number EC2019-049.

### Data availability.

The data sets generated during and/or analyzed during the study are in the Supporting Data Values XLS file.

## Author contributions

RJO, HDH, PU, EH, EL, BB, and ESW designed the research. GM, ARC, LH, IR, EP, JVS, VB, EH, EL, BB, and ESW designed the experiments. GM, ARC, LH, IR, EP, and JVS performed the experiments. GM, ARC, LH, IR, EP, JVS, VB, EH, EL, BB and ESW analyzed the data. GM, ARC, LH, IR, EL, BB, and ESW prepared the original draft. All the authors contributed to and approved the manuscript. GM and ARC have equally contributed to the completion of this manuscript, including the performance of experiments, data analysis, and writing. The order of authorship for GM and ARC is by reverse alphabetical ordering of author surnames.

## Supplementary Material

Supplemental data

Supplemental video 1

Supplemental video 2

Supplemental video 3

Supplemental video 4

Supplemental video 5

Supporting data values

## Version 1 05/07/2024

In-Press Preview

## Version 2 05/22/2024

Electronic publication
