# Supplementary material for: Differential effects of FcRn antagonists on the subcellular trafficking of FcRn and albumin
Source: JCI Insight. 2024 May 7;9(10):e176166. doi: 10.1172/jci.insight.176166 (PMC11141909; doi:10.1172/jci.insight.176166)
Supplement: Supplemental data [file jciinsight-9-176166-s226.pdf]

Supplementary materials

Differential effects of FcRn antagonists on the subcellular trafficking of FcRn and albumin

Guanglong Ma[1†], Andrew R. Crowley[1†], Liesbeth Heyndrickx[2], Ilse Rogiers[2], Eef Parthoens[3], Jolien Van Santbergen[2], Raimund J. Ober[1], Vladimir Bobkov[2], Hans de Haard[2‡], Peter Ulrichts[2], Erik Hofman[2], Els Louagie[2], Bianca Balbino[2]* and E. Sally Ward[1]*

[1] Centre for Cancer Immunology, Faculty of Medicine, University of Southampton, Southampton, SO16 6YD, United Kingdom

[2] argenx, 9052 Zwijnaarde (Ghent), Belgium

[3] VIB BioImaging Core, Center for Inflammation Research, Ghent, Belgium

[†] These authors have contributed equally to this work

[‡] Formerly an employee of argenx BV

*** Corresponding authors**

E. Sally Ward: Centre for Cancer Immunology, Faculty of Medicine, University of Southampton, Southampton, SO16 6YD, United Kingdom. Phone: +44 02381 206568. Email: E.S.Ward@soton.ac.uk

Bianca Balbino: argenx BV, Industriepark Zwijnaarde 7, Gent, Belgium 9052. Phone: +32 474 56 93 08. Email: bbalbino@argenx.com

Supplemental Methods

Size exclusion-high performance liquid chromatography

Analytical size exclusion-high performance liquid chromatography (SEC-HPLC) was performed using a Shimadzu system equipped with CMB-20A (system controller), LC-20AD (pump), and an SPD-20A UV/Vis detector. Samples were analyzed using a Yarra 3 µm SEC-3000 gel filtration column (Phenomenex, 00H-4513-E0) in DPBS at a flow rate of 0.35 mL/min.

Assays to assess IgG recycling

HEK293-hFcRn-GFP cells were seeded at 75,000 cells/well in DMEM medium (phenol red-free maintenance medium (Gibco, 2052025) containing bovine IgG-depleted FBS (1), pH 7.4 unless specified otherwise) in 24-well plates (Corning, 3524) overnight. Cells were then incubated with 3 µM FcRn antagonist, human serum IgG (hIgG, Sigma, I2511), or medium alone for 1 hour in serum-free medium at 37°C in a 5% CO_2 incubator. Subsequently, AF647-labeled hIgG (hIgG-AF647) was added to cells at a final concentration of 200 µg/mL and incubated for 1 hour. Then, cells were washed with DPBS and chased for 0 or 1 hour with 3 µM FcRn antagonist, hIgG, or medium alone in serum-free medium. Following the chase, cells were washed with ice-cold DPBS, trypsinized with 0.025% trypsin-EDTA (Gibco, 25200056), washed with ice-cold DPBS, and fixed with 3.4% paraformaldehyde (PFA, Electron Microscopy Sciences, 19200) for 15 minutes at room temperature. Cells were stored in DPBS + 1% BSA (Fisher BioReagents, BP1600-1), and the AF647 fluorescence signal was acquired using a CytoFLEX S flow cytometer and CytExpert Software (CytExpert 2.4.0.28). Data were processed using FlowJo (10.8.1).

Detection of hFcRn using Synt002 Fab

HEK293-hFcRn-GFP cells were resuspended in phenol red-free DMEM medium (pH 7.4 unless specified otherwise) with 10% FBS (bovine IgG-depleted) and seeded at 10,000 cells/dish in MatTek dishes (P35-10-C-NON) fitted with cover glasses (Electron Microscopy Sciences, no. 1.5, 22 mm diameter, 72224-01) overnight. Cells were incubated with 10 µg/mL AF555-labeled IgG1-MST-HN or HL161BK for 30 minutes at 37°C in a 5% CO_2 incubator. Cells were then washed with ice-cold DPBS

and fixed with 3.4% PFA for 15 minutes at room temperature. The cells were permeabilized with 0.25 mg/mL saponin (Sigma, S4521-10G) in DPBS for 20 minutes at room temperature followed by washing with DPBS and blocking with 4% BSA for 30 minutes at room temperature. The cells were subsequently washed with DPBS and incubated with 2 µg/mL Synt002-Fab-AF647 in DPBS + 1% BSA with 0.25 mg/mL saponin for 30 minutes at room temperature. Finally, cells were washed and stored in DPBS + 1% BSA for imaging.

Immunoblot analyses of FcRn levels

Untransfected HMEC-1 and hFcRn-GFP transiently transfected HMEC-1 cells were prepared as described in the Methods section and 250,000 cells were seeded per well of 6-well plates. The following day, the medium was replaced with fresh HAMS medium containing 50 nM ARGX-113, HL161BK, or vehicle only and cells were incubated for 24 hours at 37°C in a 5% CO_2 incubator. The cells were washed once in ice-cold PBS and lysed on ice for 30 minutes using RIPA buffer (Thermo Scientific, 89900) containing pepstatin A (Fisher Scientific, J20037.MB) and leupeptin hemisulfate (Fisher Scientific, J61188.MB). To maintain high protein concentrations in the lysate, lysis buffer was added to a well, the cells were scraped with the plunger of a 1 mL syringe, and the buffer was transferred to a replicate well to continue the process; each sample of lysate was therefore prepared from triplicate wells in this manner. Total protein concentrations were determined using a bicinchoninic acid (BCA) assay (ThermoFisher, 23225) and an Infinite M Plex plate reader (Tecan Group).

Molecular weight standards (Spectra multicolor protein ladder; ThermoFisher, 26634) and 20 µg of each lysate were run on a 4-12% acrylamide gel (GenScript, M00654) and transferred to nitrocellulose membranes (Cytiva, 10600002). The membranes were subsequently incubated in 4% BSA in PBS + 0.05% (v/v) Tween20 (Sigma, P2287-500ML), followed by overnight incubation at 4°C with a 1:1000 dilution of anti-FCGRT (Novus Biologicals, NBP1-89128) and a 1:1500 dilution of anti-GAPDH (clone GA1R) (Invitrogen, MA5-15738) in 1% BSA in PBST. Following washing, bound primary antibodies were detected using 1:4000 dilutions of anti-rabbit 680RD (Licor, 926-68073) and anti-

mouse 800CW (Licor, 926-32212) infrared secondaries. The blot was washed and imaged using an ImageQuant 800 (Cytiva).

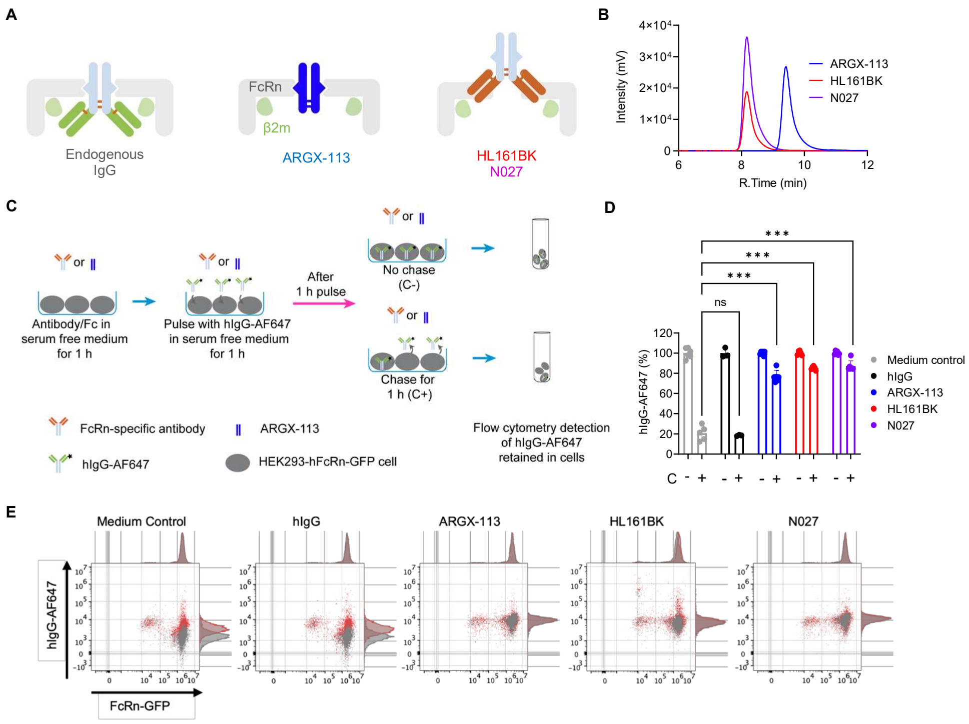

Figure S1. Characterization of FcRn antagonists

(**A**) Illustration of interaction between FcRn and endogenous IgG or engineered Fc fragment (ARGX-113) or an FcRn-specific antibody (HL161BK or N027). (**B**) Chromatograms of FcRn antagonists following size exclusion analyses using a Yarra 3 µm SEC-3000 gel filtration column. (**C-E**) Effects of FcRn antagonists on IgG recycling in HEK293-hFcRn-GFP cells. HEK293-hFcRn-GFP cells were incubated with 3 µM FcRn antagonist, human serum IgG (hIgG), or medium alone for 1 hour in serum-free medium. Subsequently, AF647-labeled hIgG (hIgG-AF647) was added to cells at a final concentration of 200 µg/mL and incubated further for 1 hour. Cells were then washed and chased for 0 (no chase, C-) or 1 hour (C+). Fluorescence levels of hIgG-AF647 following the pulse and pulse-chase were determined using flow cytometry. Data shown are normalized against the levels for no chase (C-) cells. These data are combined from two independent experiments, with triplicate samples in each experiment. Statistical analysis was performed using one-way ANOVA and significant differences are denoted as: *** $p \leq 0.001$ and ns indicates $p > 0.05$. Error bars indicate the standard deviation of the mean. **(E)** Dot plots correspond to data shown in panel D, with pulsed-only cells indicated by red coloring, and pulse-chased cells indicated by grey coloring.

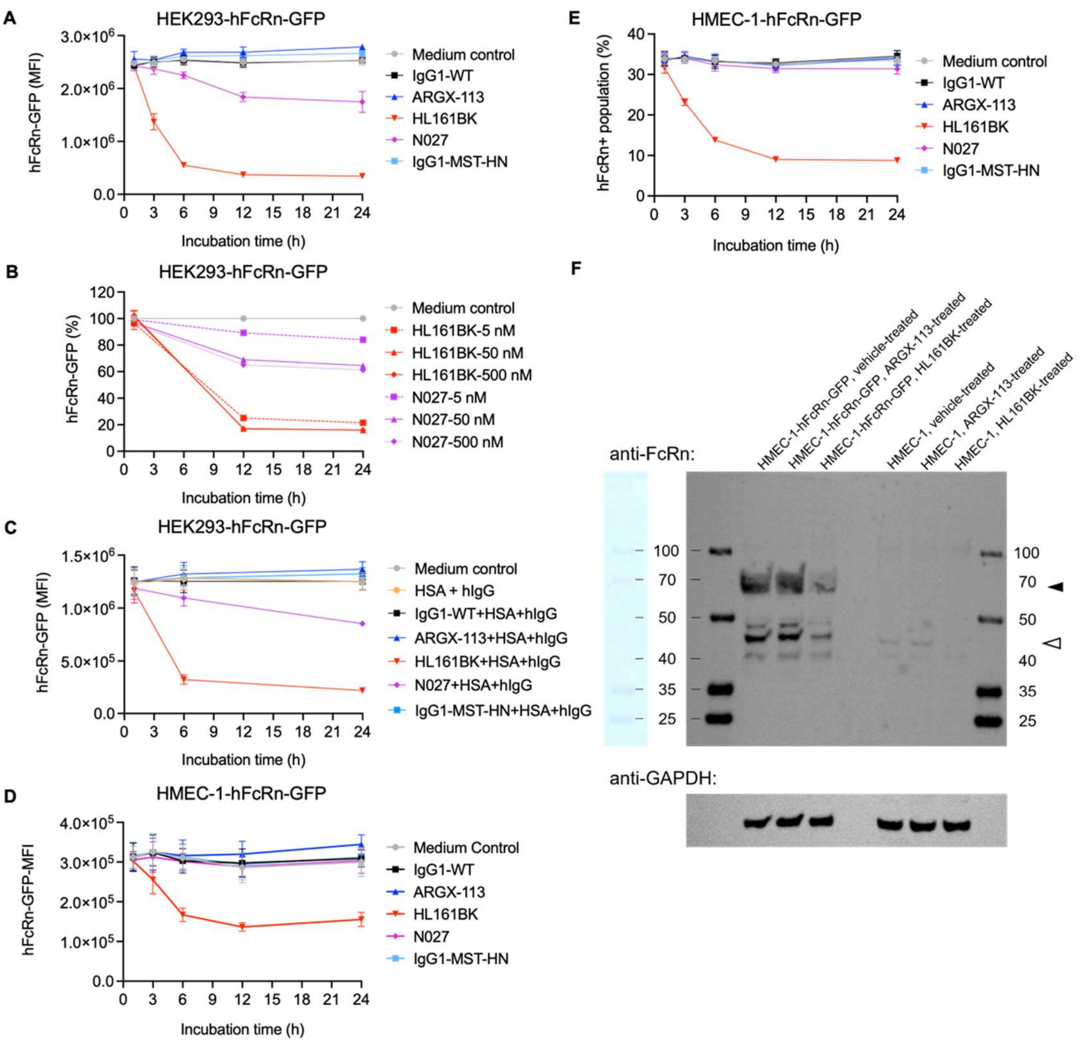

Figure S2. Analyses of human FcRn levels in antagonist-treated cell lines

(**A**) MFI for GFP levels in HEK293-hFcRn-GFP cells corresponding to Figure 1A. (**B**) HEK293-hFcRn-GFP cells were incubated with 5-500 nM anti-FcRn antagonists, and GFP fluorescence levels were normalized to medium control at each timepoint. (**C**) MFI for GFP levels in HEK293-hFcRn-GFP cells incubated with 50 nM anti-FcRn antagonists in the presence of 6 µM HSA and 2 µM hIgG, 6 µM HSA and 2 µM hIgG only, or medium control (without HSA and hIgG) for 1, 6, or 24 hours. (**D**) MFI for GFP levels in HMEC-1-hFcRn-GFP cells corresponding to Figure 1B. (**E**) hFcRn-positive ($hFcRn^+$) populations gated from HMEC-1-hFcRn-GFP cells at each timepoint, corresponding to Figure 1B and S2D. GFP fluorescence intensities were determined using flow cytometry. These data are combined from two independent experiments, with triplicate samples in each experiment, except for data shown in (**B**), which was carried out once. Error bars indicate standard deviation of the mean. (**F**) Immunoblotting analyses of the levels of endogenous hFcRn (approx. 46 kDa, denoted by open arrowhead) and transfected hFcRn-GFP (approx. 73 kDa, filled arrowhead) in HMEC-1 cells following a 24-hour treatment with 50 nM antagonists (top) and corresponding GAPDH loading controls (bottom). The two images were collected in parallel, with the FcRn detection in the short infrared (680RD) and the GAPDH detection in the long infrared (800CW) channels. These results are representative of two independent experiments.

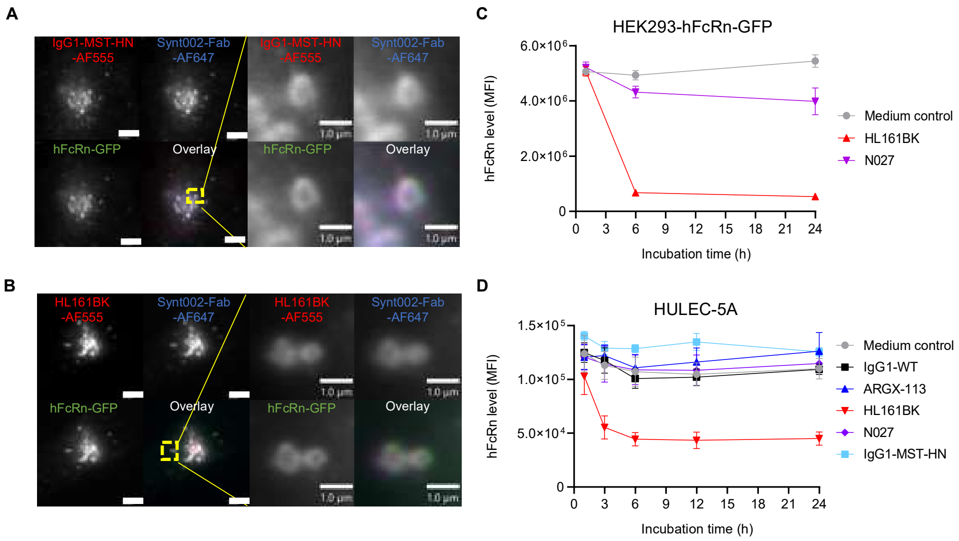

Figure S3. Characterization of Synt002-derived Fab fragment

HEK293-hFcRn-GFP cells were incubated with 10 µg/mL AF555-labeled IgG1-MST-HN (**A**) or HL161BK (**B**) for 30 minutes at 37°C with 5% CO_2. Cells were subsequently fixed, permeabilized (20 min), incubated with 4% BSA (30 min), then incubated with 2 µg/mL AF647-labeled Synt002-Fab (Synt002-Fab-AF647). Data are representative of 2 independent experiments, each consisting of 2 dishes, and at least 6 images from each dish. AF555, AF647, and GFP are pseudocolored red, blue, and green. Scale bars = 5 µm in the two left columns, 1 µm in cropped and expanded images in the two right columns (cropped and expanded regions are indicated by boxes in left column images). (**C**) Detection of hFcRn in HEK293-hFcRn-GFP cells using Synt002-Fab-AF647 following incubation with 50 nM hFcRn antagonists or medium alone for 1, 6, or 24 hours. Following antagonist treatment, cells were fixed, permeabilized and stained with 2 µg/mL Synt002-Fab-AF647. MFI levels of AF647 were determined using flow cytometry. Data are from one experiment carried out using triplicate samples. (**D**) MFI levels for Synt002-Fab-AF647 in HULEC-5A cells corresponding to Figure 1C. Data are combined from two independent experiments, with triplicate samples in each experiment. Error bars indicate standard deviation of the mean.

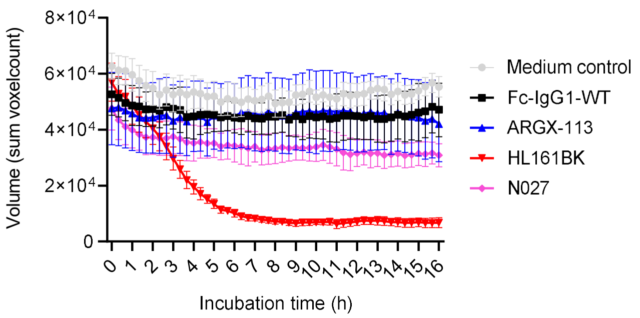

Figure S4. FcRn levels in HEK293-hFcRn-GFP cells during treatment with different FcRn antagonists

500 nM anti-FcRn antagonists or medium alone were added to the cells immediately preceding the start of a 16-hour time-lapse recording, and images were taken every 20 minutes. Graph of the volume (sum of voxels) against time for the hFcRn-GFP signal. Data represent three independent experiments. Error bars indicate standard error of the mean. Data shown correspond to Figure 1D and E.

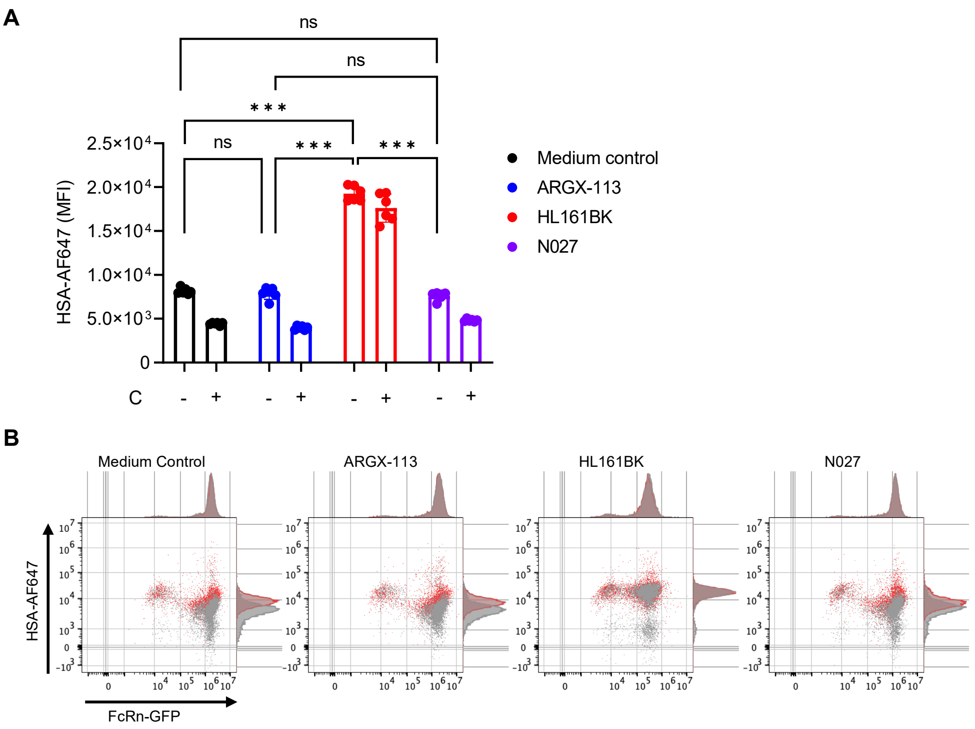

Figure S5. Effects of FcRn antagonists on recycling of HSA by HEK293-hFcRn-GFP cells
HEK293-hFcRn-GFP cells were incubated with 50 nM FcRn antagonists or medium alone for 24 hours. The cells were then starved for 2 hours in serum-free medium, pulsed with 250 µg/mL AF647-labeled HSA (HSA-AF647) in serum-free medium for 60 minutes, washed and chased in serum-free medium for 0 (no chase, C-) or 30 minutes (C+) at 37ºC in a 5% CO_2 incubator. The cell-associated HSA-AF647 levels following the indicated treatments were determined using flow cytometry. **(A)** MFI of HSA-AF647 levels in HEK293-hFcRn-GFP cells corresponding to Figure 2B**.** These data are combined from two independent experiments, with triplicate samples in each experiment. Two-way ANOVA with a log transformation was used for statistical analysis. Statistically significant differences are shown as: *** $p \leq 0.001$, ns indicates $p > 0.05$. Error bars indicate standard deviation of the mean. **(B)** Dot plots correspond to data shown in panel A, with pulsed-only cells indicated by red coloring, and pulse-chased cells indicated by grey coloring.

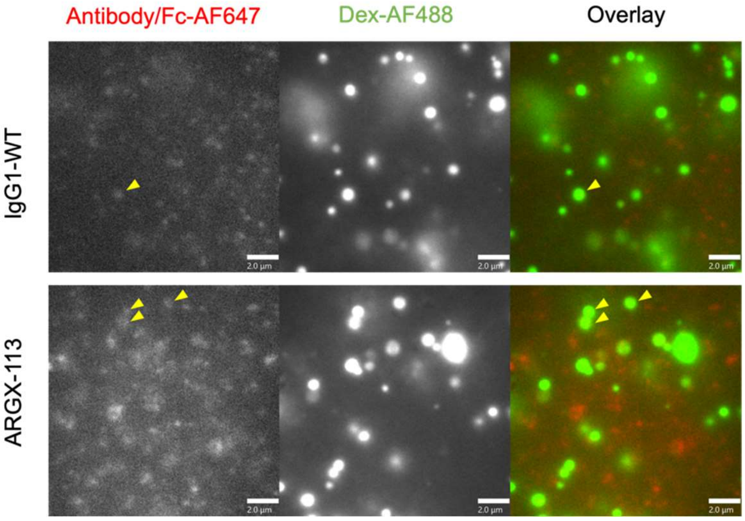

Figure S6. Lysosomal trafficking analyses in HULEC-5A cells
HULEC-5A cells were incubated with 50 nM AF647-labeled ARGX-113 or an IgG1-WT control for 24 hours. During these incubations, cells were pulsed (1 hour) and chased (6 hours) with 500 µg/mL Dex-AF488. Following incubation, cells were washed, fixed, and imaged (images correspond to Figure 6, 24 h treatment for IgG1-WT and ARGX-113). Yellow arrowheads in the panels indicate the detection of AF647-labeled ARGX-113 or IgG1-WT in dextran-positive compartments. The images correspond to those shown in Figure 6, but with adjustment to increase the AF647 signal for visualization in the left panels. Data are representative of two independent experiments, each consisting of 2 dishes for each condition, and at least 6 images for each dish. AF647 and AF488 are pseudocolored red and green, respectively. Each image represents part of a single cell and scale bars = 2 µm.

Movie S1. Movie of hFcRn-GFP fluorescence in HEK293-hFcRn-GFP cells during treatment with medium only

Movie S2. Movie of hFcRn-GFP fluorescence in HEK293-hFcRn-GFP cells during treatment with Fc-IgG1-WT

Movie S3. Movie of hFcRn-GFP fluorescence in HEK293-hFcRn-GFP cells during treatment with ARGX-113

Movie S4. Movie of hFcRn-GFP fluorescence in HEK293-hFcRn-GFP cells during treatment with HL161BK

Movie S5. Movie of hFcRn-GFP fluorescence in HEK293-hFcRn-GFP cells during treatment with N027

Movie S1-S5: images were taken every 20 minutes over the course of 16 hours, time-lapse images were processed into 8-second movies in which the maximum intensity projection was used to display the Z-stacks of the hFcRn-GFP fluorescence signal (pseudocolored green) at each timepoint. Data shown correspond to Figure 1D, E, Figure S4.

Supplementary References

1. Ober RJ, et al. Visualizing the site and dynamics of IgG salvage by the MHC class I-related receptor, FcRn. *J Immunol.* 2004;172(4):2021-9.
